# MIR17HG-miR-18a/19a axis, regulated by interferon regulatory factor-1, promotes gastric cancer metastasis via Wnt/β-catenin signalling

**DOI:** 10.1038/s41419-019-1685-z

**Published:** 2019-06-11

**Authors:** Jingsheng Yuan, Lulu Tan, Zhijie Yin, Wenzhong Zhu, Kaixiong Tao, Guobing Wang, Wenjia Shi, Jinbo Gao

**Affiliations:** 1 Department of Gastrointestinal Surgery, Union Hospital, Tongji Medical College, Huazhong University of Science and Technology, Wuhan, 430022 China; 2 Department of Paediatric Surgery, Union Hospital, Tongji Medical College, Huazhong University of Science and Technology, Wuhan, 430022 China

**Keywords:** Gastric cancer, Cell invasion, Cell signalling, miRNAs

## Abstract

MIR17HG, located on chromosome 13, is a class of Pri-miRNAs that generates six miRNAs: miR-17, miR-18a, miR-19a, miR-20a, miR-19b-1 and miR-92-1. These miRNAs are ubiquitously overexpressed in diverse tumour types and exhibit complex biological links to tumour metastasis. We demonstrated that MIR17HG-derived miR-18a and miR-19a coordinately mediate gastric cancer cell metastasis by directly inhibiting SMAD2 expression and upregulating Wnt/β-catenin signalling. Based on previous studies, we hypothesised that an investigation of MIR17HG inhibition would be beneficial to clinical gastric cancer treatment, and systematically coupled bioinformatics analyses brought interferon regulatory factor-1 (IRF-1) to our attention. We then established stable clones in gastric cancer cells containing a doxycycline-inducible IRF-1 expression system and found that the expression of IRF-1 downregulates the embedded miRNAs of MIR17HG in gastric cancer cells and inhibits gastric cancer cell metastasis by attenuating Wnt/β-catenin signalling. Further rescue assays confirmed the crucial roles of miR-18a and miR-19a in the IRF-1-mediated inhibition of Wnt/β-catenin signalling. We also demonstrated that IRF-1 binds to the transcriptional site in the MIR17HG promoter and inhibits MIR17HG expression. Moreover, IFN-γ induced the IRF-1-mediated downregulation of MIR17HG in gastric cancer cells. Our hypothesis was supported by the results of immunohistochemistry analyses of clinical gastric cancer samples, and we also demonstrated the role of IRF-1 in inhibiting MIR17HG expression and tumour metastasis in vivo. We conclude that IRF-1 inhibits gastric cancer metastasis by downregulating MIR17HG-miR-18a/miR-19a axis expression and attenuating Wnt/β-catenin signalling.

## Introduction

Gastric cancer (GC) is currently the most common upper gastrointestinal malignancy and one of the leading causes of cancer-associated mortality worldwide^1,2^. Because the early symptoms of GC are concealed and easily missed during early diagnosis or screening, the majority of GC patients are in advanced stages of the disease at their initial diagnosis^3^. Although GC treatment has matured and become increasingly diverse in recent years, the 5-year survival rate of GC patients remains relatively low, mainly owing to local lymph node and distant metastasis^4,5^. Moreover, tumour nodules that are located near critical organs or are extensively mesenteric cannot be completely excised^5,6^. Therefore, an exploration of the potential molecular mechanisms underlying metastasis will potentially benefit the prognostic evaluation and precise treatment of GC.

Numerous studies have confirmed that noncoding RNAs (ncRNAs), particularly microRNAs (miRNAs), are critical for gene expression regulation^7,8^. MIR17HG is a class of Pri-miRNAs located in the 800-base-pair region of human chromosome 13, and the sole function of this class of Pri-miRNAs is to generate six miRNAs (miR-17, miR-18a, miR-19a, miR-20a, miR-19b-1 and miR-92-1)^9^. Numerous recent studies have confirmed that MIR17HG exhibits complex links to cancer metastasis. Members of the MIR17HG family are highly expressed in the invasive tumour fronts of metastatic colorectal cancer, retinoblastoma and pancreatic cancer^10–12^. Specifically, miR-17 promotes GC migration by directly targeting early growth response 2^13^, and the inhibition of miR-17 impairs the epithelial–mesenchymal transition (EMT) in GC through the death effector domain-containing DNA-binding protein^14^. miR-20a enhances the invasiveness of human glioma stem cells by directly targeting the tissue inhibitor of metalloproteinases-2^15^. These results indicate that MIR17HG has an important role in metastasis and progression and that an exploration of the inhibitory mechanism of MIR17HG will have important clinical significance for the treatment of GC.

In this study, we investigated the function and potential mechanism of MIR17HG, including its upstream transcription factor and downstream target gene, in GC carcinogenesis. The results provide evidence showing that MIR17HG-derived miR-18a and miR-19a directly inhibit SMAD2 expression and coordinate the upregulation of Wnt/β-catenin signalling to promote GC metastasis. Subsequently, we established stable clones in GC cells with a doxycycline (Dox)-inducible IRF-1 expression system in which Dox binds to the tetracycline response elements in the expression vector and changes its conformation, activating IRF-1 protein expression^16^. Additional experiments confirmed that IRF-1 upregulation effectively inhibited the expression of MIR17HG and its downstream signals both in vivo and in vitro. Together, our study further enriches the understanding of the underlying mechanisms of MIR17HG in GC metastasis and provides further compelling evidence showing that the role of MIR17HG-miR-18a/miR-19a axis in promoting GC metastasis can be effectively inhibited by IRF-1.

## Results

### Upregulation of MIR17HG and embedded derivatives in GC

**Fig. 1:**
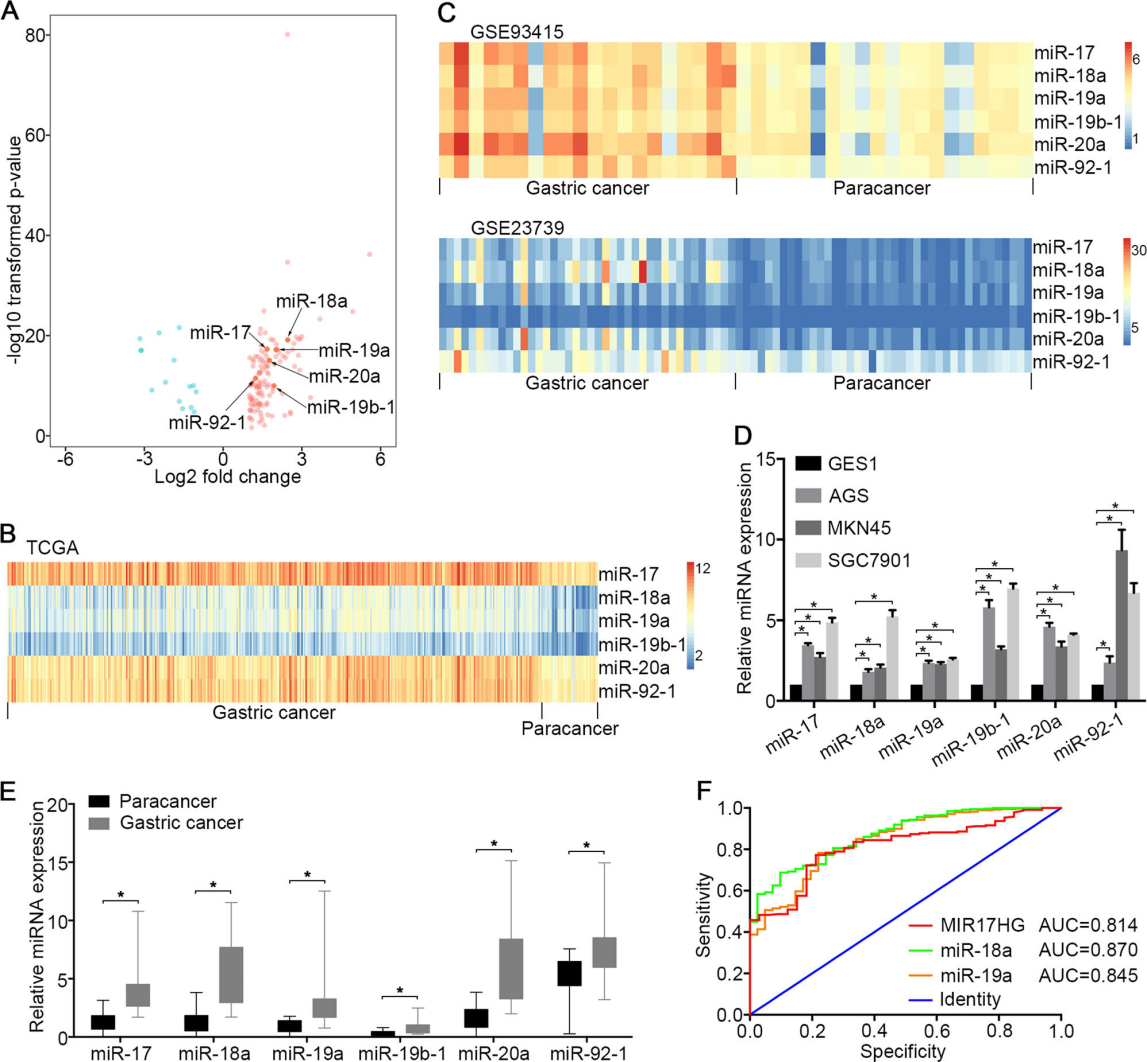
Expression profiles of miRNAs in GC cell lines and GC tissues. **a** The differentially expressed miRNAs in 387 GC samples and 41 adjacent gastric mucosal tissues are visualised in a volcano plot. The red and green colours indicate high and low expression (|log_2_(fold change) | > 1, FDR < 0.05), respectively. **b** Heatmap of six polycistronic miRNAs derived from MIR17HG in the TCGA data set. **c** Heatmap of six polycistronic miRNAs derived from MIR17HG in the GEO verification data set (GSE93415 and GSE23739). **b**, **c** the columns represent the clinical samples, while the rows show the relative expression of individual miRNAs. The red and blue colours indicate high and low expression, respectively. **d** qRT-PCR analysis of the expression of six polycistronic miRNAs derived from MIR17HG in GC cell lines (AGS, MKN45 and SGC7901) and a normal gastric epithelial cell line (GES1). **e** qRT-PCR analysis of the expression of six polycistronic miRNAs derived from MIR17HG in 10 pairs of cancerous and adjacent tissues harvested from patients with GC. **d**, **e** U6 snRNA served as the internal control. *N* = 3 independent experiments performed in triplicate. *P < 0.05, as demonstrated by paired Student’s *t* test. The data are presented as the means ± standard deviations (SDs). **f** Receiver operating characteristic (ROC) curve of MIR17HG, miR-18a and miR-19a among GC patients

A miRNA-Seq analysis of the Cancer Genome Atlas (TCGA) database showed that 126 and 15 transcripts were upregulated and downregulated, respectively, in GC compared with adjacent gastric mucosal tissues (Fig. 1a). Notably, the upregulated transcripts included miR-17, miR-18a, miR-19a, miR-20a, miR-19b-1 and miR-92-1 (Fig. 1b). We further validated this result using the GSE93415 and GSE23739 data sets and confirmed that these six miRNAs were upregulated in GC compared with adjacent tissues (Fig. 1c). A quantitative reverse transcription PCR (qRT-PCR) analysis confirmed that six endogenous miRNAs were more highly expressed in the GC cell lines MKN45, AGS and SGC7901 than in the gastric epithelial cell line GES1 (Fig. 1d). MIR17HG encodes miR-17, miR-18a, miR-19a, miR-20a, miR-19b-1 and miR-92–1 in the third intron of an ~7-kb primary transcript of chromosome 13 known as C13orf25 (Supplementary Fig. S1A)^9^. Therefore, we conducted a differential expression analysis of the RNA-Seq data and found that the MIR17HG transcript was significantly upregulated in GC compared with its expression in adjacent tissues (Supplementary Fig. S1B). Moreover, an analysis of the TCGA GC data repository revealed that the expression of these six miRNAs was tightly correlated with MIR17HG expression (Supplementary Fig. S1C). These findings prompted us to further explore the function of MIR17HG and its embedded derivatives in the development of malignant GC.

### Clinical correlation of MIR17HG and embedded derivatives in GC

To assess whether the six miRNAs were overexpressed in cells other than those described above, we first quantitatively analysed their expression in 20 pairs of GC and paracancerous samples and observed that miR-17, miR-18a, miR-19a, miR-20a, miR-19b-1 and miR-92–1 were more highly expressed in GC tissues than in adjacent tissues (Fig. 1e). Using the TCGA database, a significant association for the expression of MIR17HG and its embedded derivatives with patient overall survival was not observed (data not shown). However, MIR17HG, miR-18a and miR-19a may be important diagnostic predictors of GC, as demonstrated by an area under the receiver operating characteristic (ROC) curve (AUC) > 0.80 (Fig. 1f). In addition, the trend towards a difference in lymph node metastasis between patients with different expression levels of MIR17HG was significant (Supplementary Table S1). The high expression of miR-18a and miR-17 was significantly associated with lymph node metastasis and distant metastasis, whereas the high expression of miR-19a and miR-20a was significantly associated with lymph node metastasis (Supplementary Table S2 and S3). However, a correlation between the expression of miR-19b-1 and miR-92–1 and lymph node metastasis or distant metastasis was not observed (Supplementary Table S3). As the mechanisms by which miR-17 and miR-20a promote tumour metastasis have been previously reported^13–15^, our goal was to further explore the potential functions of miR-18a and miR-19a in GC metastasis.

### miR-18a and miR-19a promote Wnt/β-catenin signalling by repressing SMAD2

**Fig. 2:**
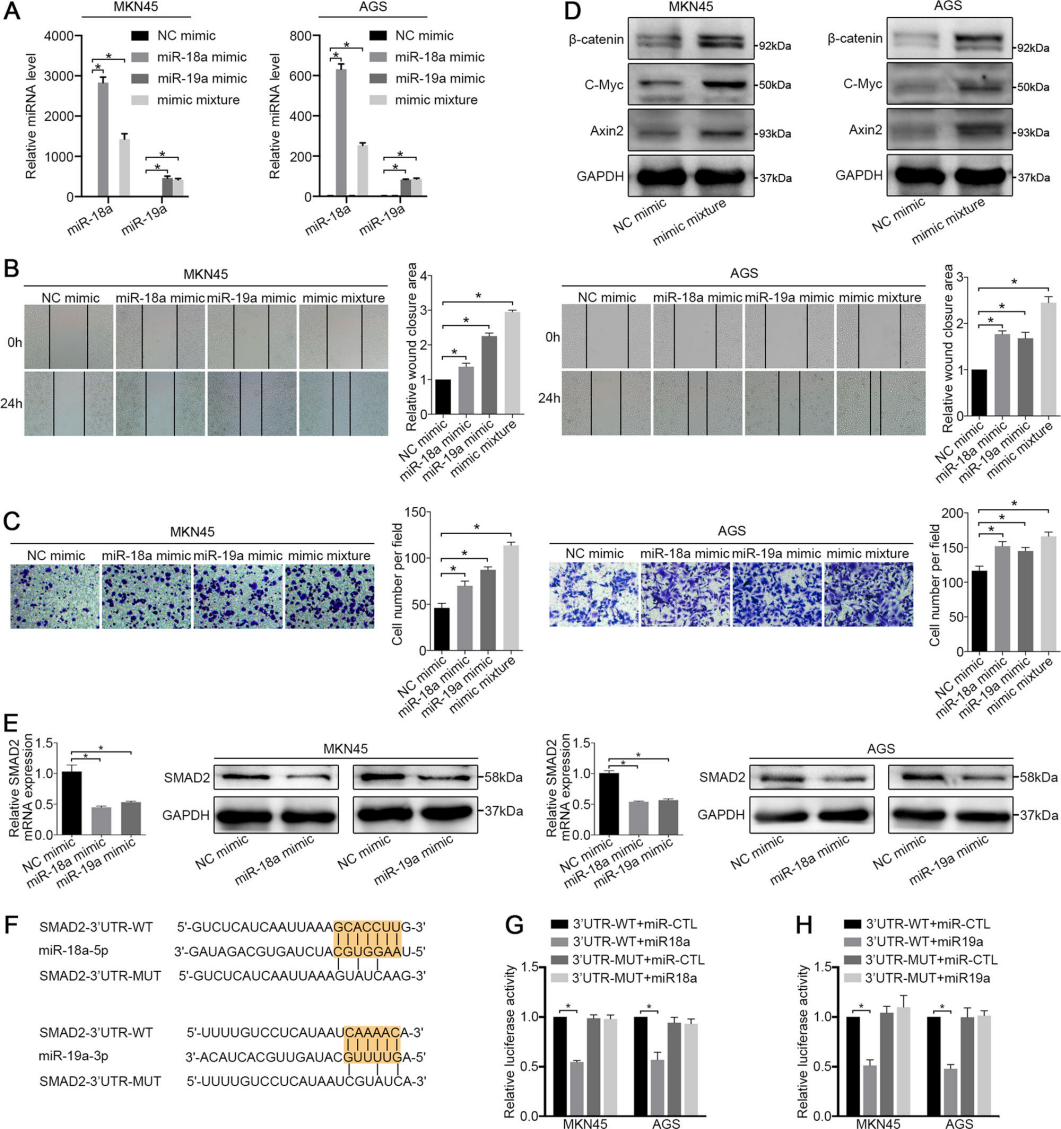
miR-18a and miR-19a cooperate to drive GC cell metastasis viaWnt/β-catenin signalling pathways. **a** At 48 hours after transfection, the expression levels of miR-18a and miR-19a in the MKN45 and AGS cell lines were examined by qRT-PCR. U6 snRNA served as the internal control. **b** Wound-healing assay and **c** migration assay of MKN45 and AGS cells treated with an NC mimic, a miR-18a mimic, a miR-19a mimic and a mimic mixture. **d** Western blot analysis of β-catenin, C-Myc and Axin2 in MKN45 and AGS cells treated with an NC mimic and a miR-18a/19a mimic mixture. **e** The mRNA and protein levels of SMAD2 in cells treated with miR-18a mimic, miR-19a mimic and NC mimic were examined. **f** Predicted miR-18a and miR-19a-binding sites in the 3’ UTRs of human SMAD2. **g**, **h** Dual luciferase assays of SMAD2 that were predicted to be regulated by miR-18a or miR-19a. All the above experiments were independently performed in triplicate (*N* = 3). The data **a**, **b**, **c**, **e**, **g** and **h** represent the means ± SDs. *P < 0.05, as demonstrated by paired Student’s *t* test

To elucidate the roles of miR-18a and miR-19a in GC metastasis, we first investigated the overexpression status of these miRNAs, and a qRT-PCR analysis showed that the expression of miR-18a, miR-19a and their mimic mixture after the transfection was significantly increased in both cell lines (Fig. 2a). A wound-healing assay showed that both the individual and mixture transfections resulted in a smaller gap between the scorings in the mimic-treated group than in the NC mimic group after 24 hours, and when introduced simultaneously, the miR-18a/miR-19a mixture conferred the strongest prohealing effect (Fig. 2b). In cell migration assays, the exogenous upregulation of miR-18a/19a expression significantly increased the number of migrated cells, whereas the simultaneous introduction of the mimic significantly increased the number of migrated cells compared with that obtained with when the mimic was introduced alone (Fig. 2c). Moreover, the knockdown of miR-18a and miR-19a resulted in decreased cell migration and wound-healing efficiency compared with the NC group (Supplementary Fig. S2A–S2C).

We then investigated the potential roles of miR-18a and miR-19a in affecting GC proliferation and cell cycle regulation. Our results revealed that the mimic groups exhibited higher proliferation and colony formation efficiency than the NC groups (Supplementary Fig. S3A and S3B), whereas the knockdown of miR-18a and miR-19b had the opposite effect on cell proliferation (Supplementary Fig. S3C and S3D). In addition, as shown in Supplementary Fig. S3E, changes in the cell cycle were not observed after miR-18a and miR-19b were knocked down in GC cells.

We subsequently performed a bioinformatics analysis of the potential targets of six miRNAs derived from MIR17HG (Supplementary Table S4)^17,18^ and performed a functional enrichment analysis, the results of which revealed that the Wnt/β-catenin signalling pathway was enriched in the putative targets (Supplementary Table S5)^19,20^. Subsequently, a western blot analysis after the overexpression of miR-18a and miR-19a revealed that the expression of β-catenin, C-Myc and Axin2 in the Wnt/β-catenin signalling pathway was significantly increased (Fig. 2d), whereas the knockdown of miR-18a and miR-19b suppressed the expression of β-catenin, C-Myc and Axin2 (Supplementary Fig. S4A). In addition, the EMT is a developmental process that is important for wound-healing and cancer metastasis^21^. We therefore examined changes in the expression of E-cadherin, N-cadherin and vimentin by western blotting and observed no significant differences in the levels of these proteins between before and after exogenous miRNA overexpression (Supplementary Fig. S4B). Subsequently, as shown in Supplementary Tables S4 and S5, we identified seven potential target genes of miR-18a and miR-19a enriched in the Wnt/β-catenin signalling pathway (Supplementary Fig. S4C). Because the expression of SMAD family member 2 (SMAD2) was most significantly reduced, as demonstrated by qRT-PCR analysis (Supplementary Fig. S4D), and SMAD2 has been confirmed to be a negative regulator of β-catenin^22^, we identified SMAD2 as a target gene for further study. The overexpression of miR-18a and miR-19a decreased the mRNA and protein levels of SMAD2 in both cell lines (Fig. 2e). Through luciferase reporter assays, we further confirmed that SMAD2 is a direct target of miR-18a and miR-19a in both MKN45 and AGS cells, and the repression of SMAD2 was rescued by reporter constructs containing the corresponding mutant SMAD2 3’-UTR (Fig. 2f–h). These results suggest that miR-18a and miR-19a promote GC metastasis by directly transcriptionally repressing SMAD2 expression and thereby increasing Wnt/β-catenin signalling.

### IRF-1 downregulates MIR17HG expression

To explore the potential mechanisms through which miR-18a and miR-19a are regulated in GC, we investigated the transcriptional regulation of the host gene MIR17HG. Possible transcription factors containing binding sites in the MIR17HG promoter were mapped using PROMO^23,24^ and cross-aligned with the JASPAR database (Supplementary Table S6)^25^. Among these transcription factors, IRF-1 is a nuclear transcription regulator involved in the regulation of interferon expression and of both innate and acquired immunity and lymphocyte differentiation^26^. However, the role of IRF-1 in GC metastasis has not been reported. Public databases indicate that IRF-1 expression is significantly decreased in multiple tumour tissues, including GC, compared with that in nontumour adjacent tissues (Supplementary Fig. S5A). More importantly, an analysis of TCGA data revealed that IRF-1 expression was significantly negatively correlated with MIR17HG, miR-18a and miR-19a expression (Supplementary Fig. S5B and S5C). A qRT-PCR analysis of GC tissues repeatedly confirmed a negative correlation of miR-18a and miR-19a with IRF-1 expression (Supplementary Fig. S5D).

**Fig. 3:**
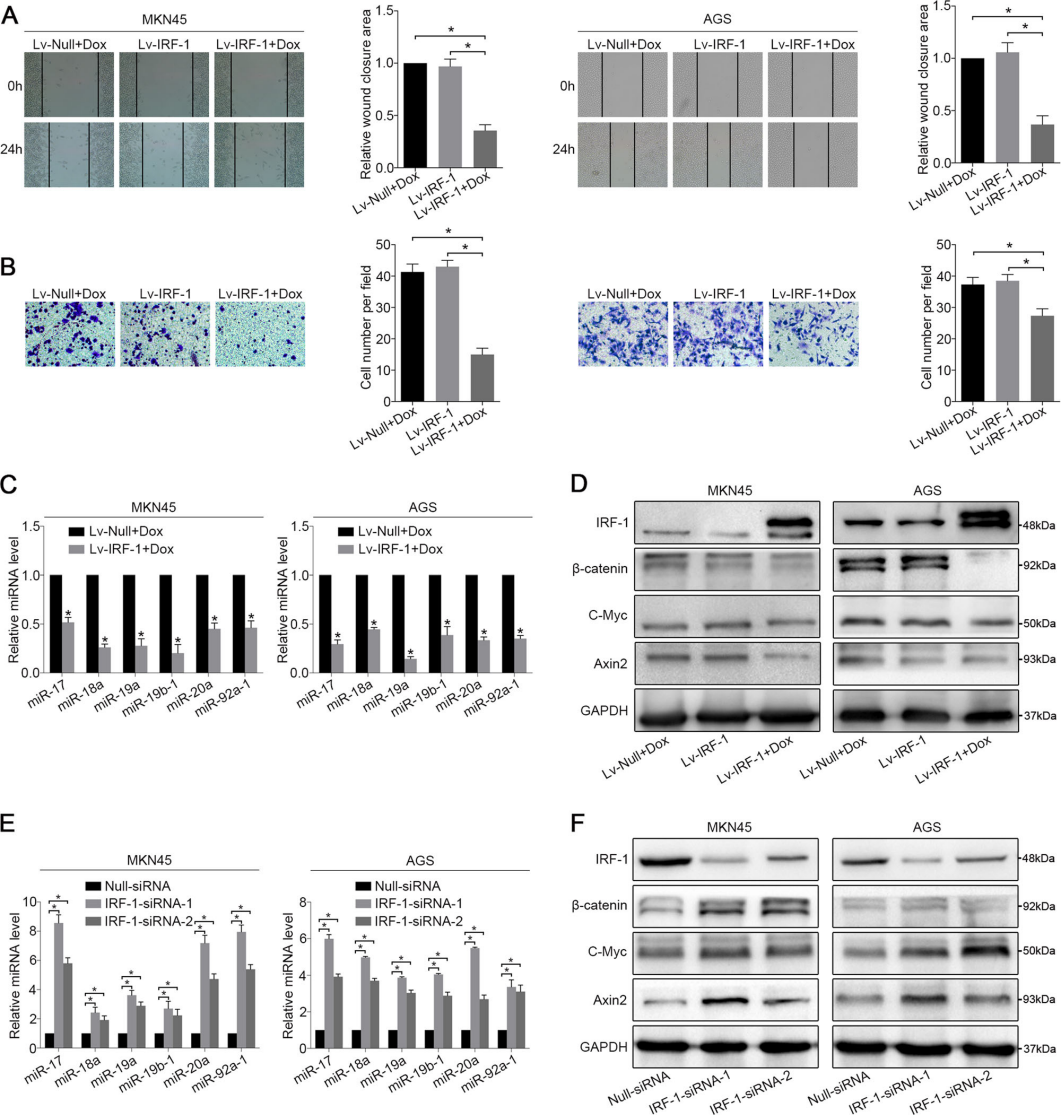
IRF-1 regulates MIR17HG expression. **a** Wound-healing and **b** migration assays of Lv-IRF-1 Dox-treated, Lv-IRF-1 Dox-untreated, and Lv-Null Dox-treated MKN45 and AGS cells were performed. **c** After 48 hours of Dox induction, the expression of six polycistronic miRNAs derived from MIR17HG in the MKN45 and AGS cell lines transfected with Lv-IRF-1 and Lv-Null was analysed by qRT-PCR. **d** After 48 hours of Dox induction, IRF-1, β-catenin, C-Myc and Axin2 expression in the MKN45 and AGS cell lines transfected with Lv-IRF-1 and Lv-Null was analysed by western blot analysis. **e** qRT-PCR analysis of the expression of six polycistronic miRNAs derived from MIR17HG in the MKN45 and AGS cell lines after IRF-1 knockdown. **f** Western blot analysis of IRF-1, β-catenin, C-Myc and Axin2 expression in MKN45 and AGS cell lines after IRF-1 knockdown. All the above experiments were independently performed in triplicate (*N* = 3). *P < 0.05, as determined by paired Student’s *t* test. The data **a**, **b**, **c** and **e** are presented as the means ± SDs

To further confirm the role of IRF-1, the GC cell lines MKN45 and AGS were infected with Lv-IRF-1 and the empty vector Lv-Null, respectively. After Dox induction, Lv-IRF-1 substantially inhibited the wound-healing and migration abilities of MKN45 and AGS cells compared with those of the controls (Fig. 3a, b), whereas the overexpression of IRF-1 appeared to have no significant effect on cell proliferation (Supplementary Fig. S5E and S5F). Furthermore, Dox-induced IRF-1 overexpression significantly inhibited the expression of six miRNAs derived from MIR17HG, and the expression of these miRNAs was not inhibited by Lv-Null. In addition, miR-18a and miR-19a were downregulated by >50% of their original expression levels in both cell lines (Fig. 3c). We then examined the effects of IRF-1 on the expression of members of the Wnt/β-catenin signalling pathway using Lv-IRF-1-infected GC cells. As shown in Supplementary Fig. S6A and 3D, IRF-1 substantially increased the protein expression of SMAD2 and reduced that of β-catenin, C-Myc and Axin2. The opposite effect was observed in GC cell lines after IRF-1 knockdown. The suppression of IRF-1 expression increased the wound-healing and migration of GC cells (Supplementary Fig. S6B and S6C), which was similar to the effect of miR-18a and miR-19a overexpression. A qRT-PCR analysis showed that knockdown of IRF-1 upregulated the levels of these six miRNAs by more than two-fold (Fig. 3e). The protein levels of β-catenin, C-Myc and Axin2 in IRF-1-siRNA-infected GC cells were also significantly increased compared with those observed in the Null-siRNA control cells (Fig. 3f). To further verify the tissue correlation between IRF-1 and Wnt/β-catenin signalling, we divided the GC samples into groups with low and high miR-18a/19a expression. An immunohistochemistry (IHC) analysis showed that IRF-1 was negatively correlated with β-catenin and C-Myc (Supplementary Fig. S6D).

### IFN-γ inhibits MIR17HG expression in GC cells

**Fig. 4:**
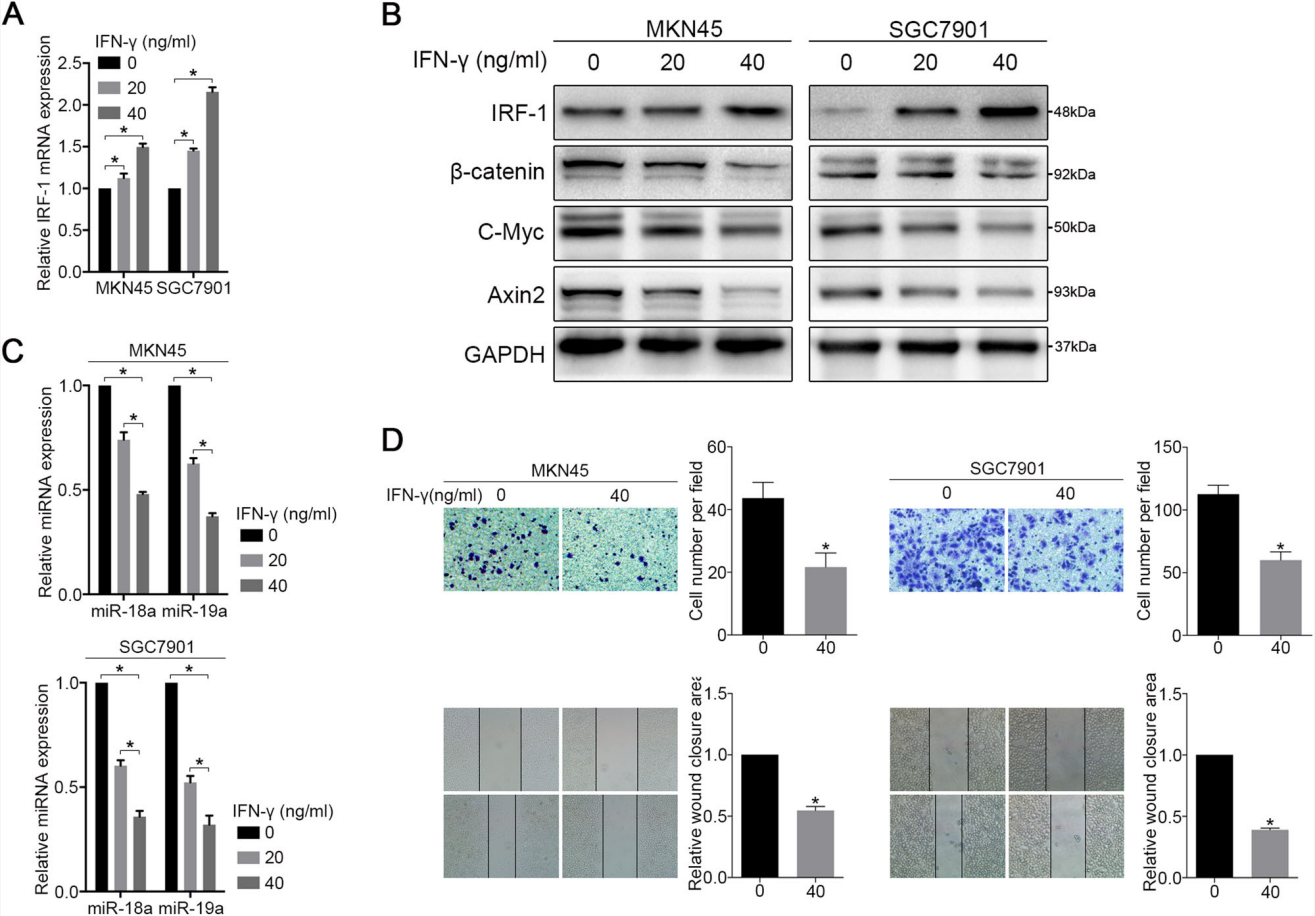
IFN-γ inhibits MIR17HG expression through IRF-1. MKN45 and SGC7901 cells were treated with the indicated amount of IFN-γ for 48 hours, and **a** the mRNA expression of IRF-1 was determined by qRT-PCR. **b** The expression of IRF-1, β-catenin, C-Myc and Axin2 was analysed by western blotting. **c** the expression of miR-18a and miR-19a was analysed by qRT-PCR, and **d** migration and wound-healing assays were performed. Three independent experiments were performed in triplicate (*N* = 3). *P < 0.05, as demonstrated by paired Student’s *t* test. The data in **a**, **c** and **d** are presented as the means ± SDs

Our previous studies demonstrated that IFN-γ is one of the strongest inducers of IRF-1 expression in cancer cells^27^. In the present study, we first confirmed the expression of Stat1 in select cell lines that did not prevent the induction of IRF-1 by IFN-γ (Supplementary Fig. S6E)^28^. We then demonstrated that IFN-γ increases IRF-1 expression in MKN45 and SGC7901 cells in a dose-dependent manner (Fig. 4a, b). The expression of miR-18a and miR-19a was inhibited by at least twofold in MKN45 and SGC7901 cells after treatment with 40 ng of IFN-γ compared with the levels in the non-treated group (Fig. 4c). Furthermore, the protein expression of β-catenin, C-Myc and Axin2 was also correspondingly reduced (Fig. 4b), and treatment with 40 ng of IFN-γ significantly inhibited the wound-healing and migration abilities of MKN45 and SGC7901 cells compared with those of the controls (Fig. 4d). However, IFN-γ did not clearly increase the expression of IRF-1 in non-Stat1-expressing AGS cells (Supplementary Fig. S6F).

### IRF-1 inhibits MIR17HG promoter activity

**Fig. 5:**
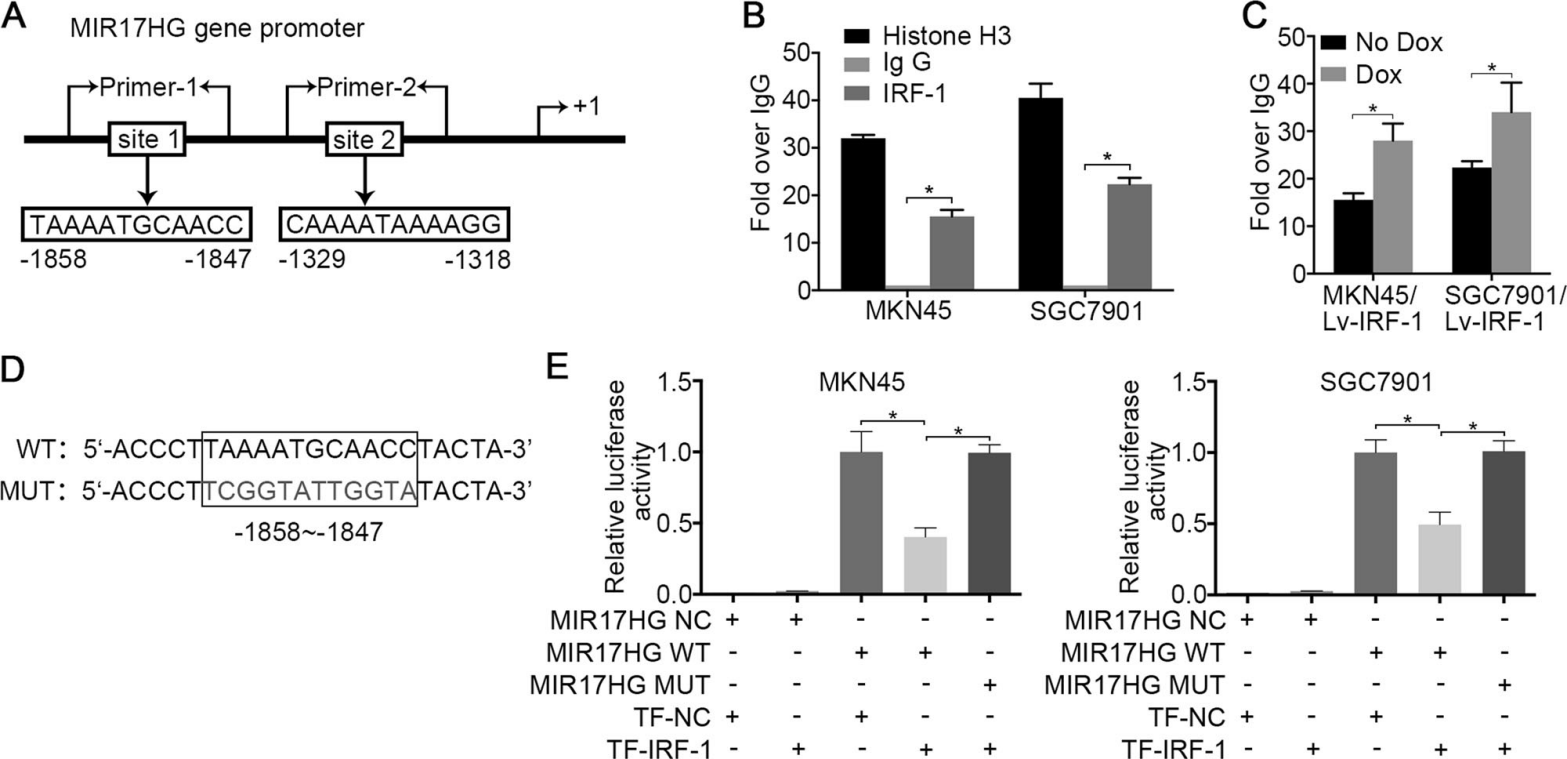
IRF-1 suppresses MIR17HG promoter activity. **a** Two putative IRF-1 transcriptional binding sites in the MIR17HG gene promoter region. **b** ChIP assays of MKN45 and SGC7901 cells were performed to characterise the recruitment of IRF-1 to the MIR17HG gene promoter. **c** MKN45/Lv-IRF-1 and SGC7901/Lv-IRF-1 cells were treated with 4 μg/ml Dox for 24 hours, and ChIP analyses were performed to assess the binding of IRF-1 to the MIR17HG gene promoter. **d** Schematic diagram of the reporter constructs of the wild-type (WT) and mutant (MUT) MIR17HG promoter binding site-1 fragment. **e** The indicated MIR17HG reporter constructs were co-transfected into MKN45 and SGC7901 cells with IRF-1 plasmids (TR-IRF-1) or normal control plasmids (TR-NC), and the luciferase activities were then measured and analysed. Three independent experiments were performed in triplicate (*N* = 3). *P < 0.05, as determined by paired Student’s *t* test. The data **b**, **c** and **e** are presented as the means ± SDs

As described above, two putative IRF-1 transcriptional binding sites in the MIR17HG gene promoter region were identified by integrating the results of the online bioinformatics analysis (Fig. 5a). A chromatin immunoprecipitation (ChIP) assay was performed to analyse the recruitment of IRF-1 by the MIR17HG promoter in MKN45 and SGC7901 cells. As shown in Fig. 5b, based on the qRT-PCR reads and the ChIP analysis results, the immunoprecipitation of IRF-1 with fragmented chromatin revealed increased PCR production using specific primers surrounding the putative tandem binding site-1 but not site-2. Following Dox treatment, a significant increase in PCR amplification was observed in Lv-IRF-1 cells, indicating an increase in the binding of IRF-1 to binding site-1 (Fig. 5c). We subsequently investigated whether IRF-1 inhibits the transcriptional activity of the MIR17HG promoter through a luciferase reporter. Smaller fragments containing wild-type and mutant site-1 sequences were cloned into the MIR17HG vector (Fig. 5d). Forced IRF-1 expression decreased the luciferase activity of cells transfected with a vector containing the wild-type site-1 sequence, and this suppression was reversed by mutation of the target sequences of MIR17HG (Fig. 5e). These results indicate that the wild-type site-1 sequence of the MIR17HG promoter is largely responsible for the inhibition of its transcription by IRF-1.

### miR-18a and miR-19a reverse the inhibitory effect of IRF-1

**Fig. 6:**
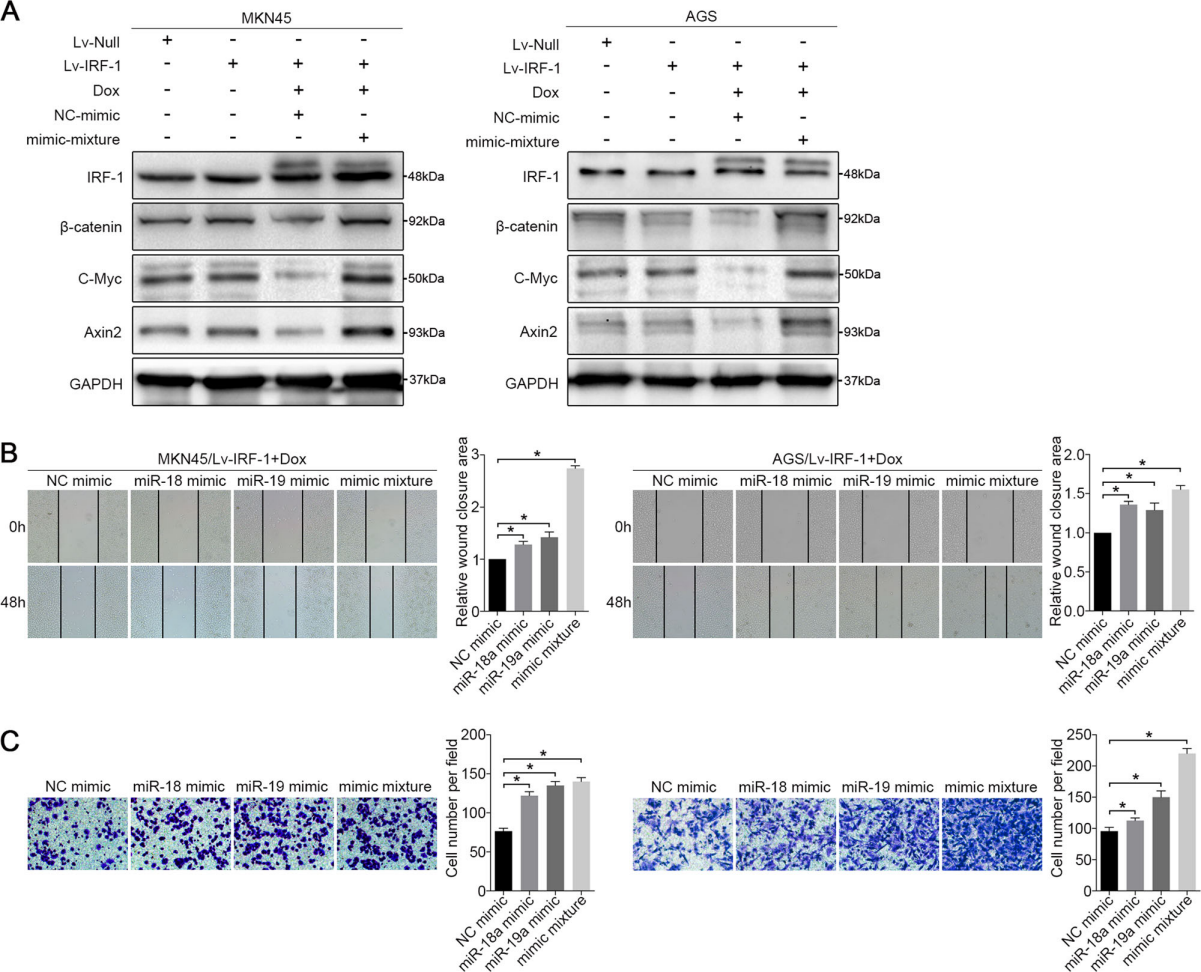
miR-18a and miR-19a reverse the inhibitory effect of IRF-1. **a** After 48 hours of Dox induction, the expression levels of IRF-1, β-catenin, C-Myc and Axin2 in MKN45/Lv-IRF-1 and AGS/Lv-IRF-1 cells treated with a mimic mixture and an NC mimic compared with those in MKN45 and AGS cell lines transfected with Lv-IRF-1 and Lv-Null, respectively, were analysed by western blotting. **b** Wound-healing assay and **c** migration assay of MKN45/Lv-IRF-1 and AGS/Lv-IRF-1 cells treated with an NC mimic, a miR-18a mimic, a miR-19a mimic and a mimic mixture. All the above experiments were independently performed in triplicate (*N* = 3). *P < 0.05, as determined by paired Student’s *t* test. The data in **b** and **c** are presented as the means ± SDs

To further investigate the roles of miR-18a and miR-19a in the IRF-1-mediated inhibition of Wnt/β-catenin signalling in GC cells, we established a “rescue” assay to investigate the effect of IRF-1 in the presence of miR-18a and miR-19a overexpression. MKN45 and AGS cells were cultured in a medium containing 4 µg/ml Dox. After 48 hours of stable IRF-1 overexpression, we delivered the mimic mixture and the NC mimic into the two cell lines. We first verified that the expression of β-catenin, C-Myc and Axin2 in Dox-induced IRF-1-overexpressing cells was significantly downregulated compared with their expression in the Lv-Null- and Lv-IRF-1-transfected cells, and their expression levels were not affected by the NC mimic. Thereafter, a western blot analysis indicated that IRF-1-inhibited β-catenin, C-Myc and Axin2 expression was reproducibly increased after transfection with the mimic mixture (Fig. 6a). Notably, unlike the results in untransfected Lv-IRF-1 cells, 48 hours were required to observe obvious trends in the wound-healing and cell transwell assays when miR-18a and miR-19a were individually or simultaneously transfected into the Dox-induced IRF-1-overexpressing cells. The wound-healing and transwell experiments demonstrated that the constitutive expression of miR-18a and miR-19a partially restored the migratory abilities of MKN45/Lv-IRF-1 and AGS/Lv-IRF-1 cells (Fig. 6b, c). Collectively, our data suggest that miR-18a and miR-19a are required for the IRF-1-mediated inhibition of Wnt/β-catenin signalling in GC.

### In vivo IRF-1-mediated inhibition of MIR17HG

**Fig. 7:**
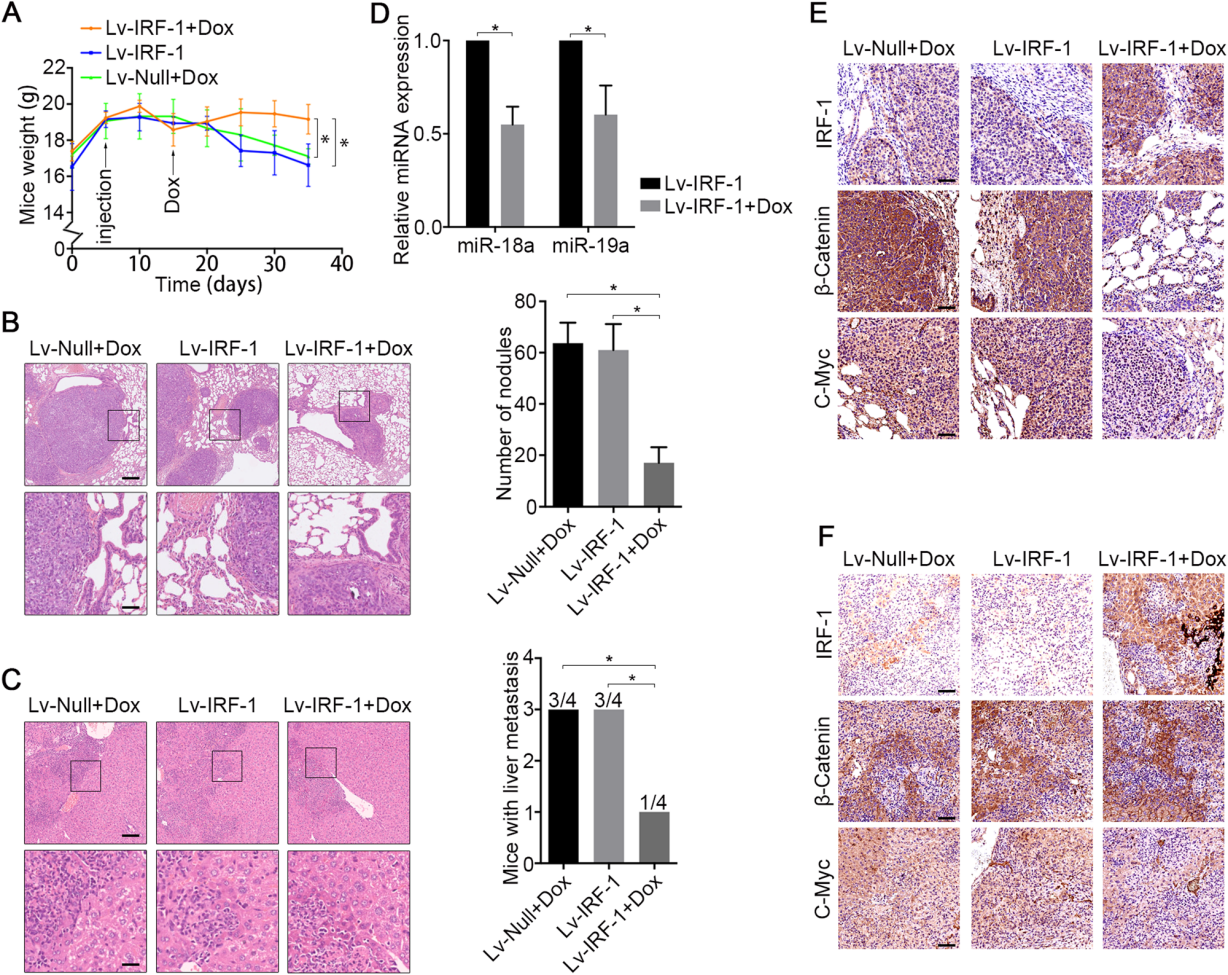
IRF-1 inhibits tumour metastases in vivo by affecting MIR17HG expression and Wnt/β-catenin signalling. **a** Average body weights of mice per group throughout the experiment. **b** H&E staining of lung tissue and number of nodules per mouse with lung metastases in each group. Scale bars, 200 μm (above) and 50 μm (below). **c** H&E staining of liver tissue and number of mice with liver metastases in each group. Scale bars, 100 μm (above) and 25 μm (below). **d** The lung metastasis nodules of the Lv-IRF-1 and Lv-IRF-1 + Dox groups were carefully stripped, and the expression levels of miR-18a and miR-19a were analysed by qRT-PCR. *P < 0.05, as determined by two-tailed unpaired Student’s *t* test. The data in **a**, **b**, **c** and **d** are presented as the means ± SDs. **e** Lung and **f** liver tissue, the expression of IRF-1, β-catenin and C-Myc were analysed by immunohistochemistry. *N* = 4 biological replicates of each experiment. Scale bars, 100 μm

Given the anti-MIR17HG-mediated metastatic effects of IRF-1 in vitro, we subsequently questioned whether the inhibition of IRF-1 affects tumour metastasis in vivo. Because MIR17HG was expressed at higher levels in SGC7901 cells than in MKN45 or AGS cells (Fig. 1d), we prioritised this cell line in our studies. The animal body weight was measured every 5 days as an indicator of tumour cachexia. After 10 days of consuming water supplemented with 4 mg/ml Dox, the body weights of the mice in the Lv-IRF-1 and Lv-Null + Dox groups were significantly decreased compared with those of the mice in the Lv-IRF-1 + Dox treatment group (Fig. 7a). A post-mortem examination at day 30 showed obvious tumour metastasis lesions in the mouse lungs and liver. All the mice exhibited secondary lung metastasis, whereas the mice in the Lv-IRF-1 + Dox treatment group exhibited fewer lung metastases than the mice in the other groups, as demonstrated by haematoxylin and eosin (H&E) staining (Fig. 7b). Three of the mice in the Lv-IRF-1 and Lv-Null + Dox groups showed liver metastasis, but only one of four Lv-IRF-1 + Dox-treated mice showed liver metastasis (Fig. 7c). To further evaluate the anti-metastatic effect of IRF-1 expression, the metastasised tumour nodules in lung tissues were carefully dissected. A qRT-PCR analysis showed that the expression of miR-18a and miR-19a in the Lv-IRF-1 + Dox group was significantly lower than that in the Lv-IRF-1 group (Fig. 7d). Further IHC analysis showed that the tumours in the Lv-IRF-1 group treated with Dox exhibited significantly increased IRF-1 expression and significantly reduced β-catenin and C-Myc levels compared with those in the Lv-IRF-1 and Lv-Null + Dox groups (Fig. 7e, f). This finding indicates that high IRF-1 expression inhibits experimental tumour metastases by affecting miR-18a and miR-19a expression and Wnt/β-catenin signalling.

## Discussion

**Fig. 8:**
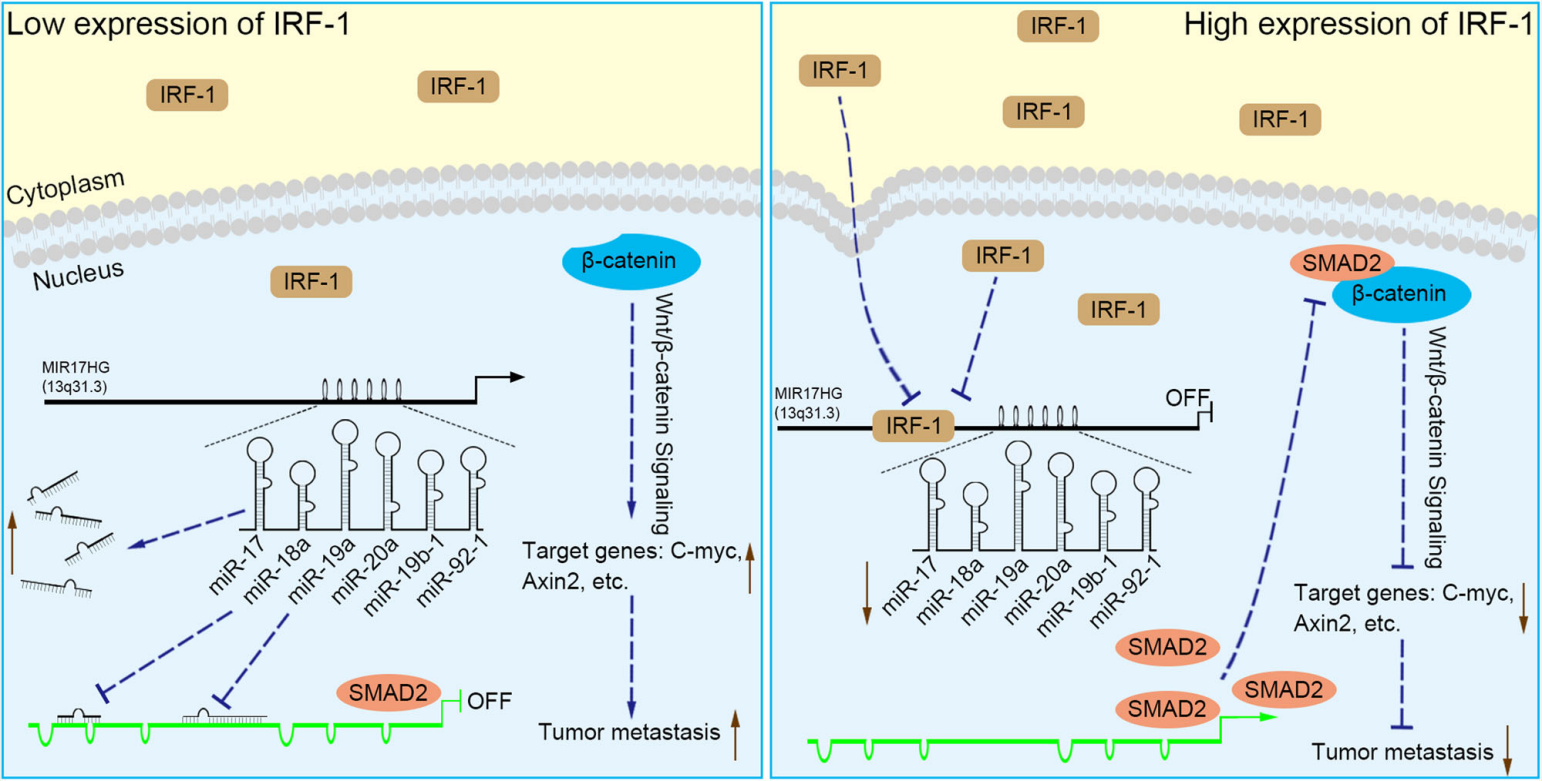
Schematic diagram of the results of our study. When IRF-1 expression in GC is low, large numbers of miR-17, miR-18a, miR-19a, miR-20a, miR-19b-1 and miR-92–1 are derived from MIR17HG, of which miR-18a and miR-19a decrease SMAD2 protein expression by inhibiting its promoter activity, attenuating its inhibitory effect on β-catenin. The increased expression of Wnt/β-catenin signalling elements, such as C-Myc, Axin2, etc., increases the metastasis of GC. When IRF-1 expression is increased, MIR17HG promoter activity is inhibited and the expression of miRNAs from MIR17HG is reduced. Subsequently, the inhibitory effect of miR-18a and miR-19a on SMAD2 promoter is attenuated. The increased expression of SMAD2 leads to it binding β-catenin and inhibiting its activity, thereby attenuating Wnt/β-catenin signalling and inhibiting GC metastasis

Expression profiling studies have confirmed that MIR17HG is ubiquitously overexpressed in diverse tumour types, including both haematopoietic malignancies and solid tumours, such as those derived from stomach, prostate and lung tissue^29^. Further studies found that increased MIR17HG expression is associated with a poor prognosis in patients with diverse types of tumours^30,31^, and single-nucleotide polymorphisms of MIR17HG are associated with a risk of breast cancer development^32^. In our study, although an obvious correlation between MIR17HG expression and the prognosis of GC patients was not observed, an analysis of the TCGA data repository revealed a correlation between the high expression of MIR17HG and its embedded miR-18a/19a and lymph node metastasis. Moreover, MIR17HG was found to be a potential predictive biomarker of GC. These data support the hypothesis that MIR17HG drives GC metastasis and progression. By systematically coupling bioinformatics analyses, in vitro and in vivo experiments, and clinical sample data, we elucidated the potential mechanisms through which MIR17HG-miR-18a/19a axis induces GC invasive metastasis, and more importantly, we demonstrated that the role of MIR17HG in driving GC metastasis could be inhibited by IRF-1 (Fig. 8).

Given that the sole purpose of MIR17HG is to facilitate the expression of its embedded miRNAs^9^, we first explored the potential mechanisms through which MIR17HG promotes GC metastasis. Combined with previous studies, we selected miR-18a and miR-19a for further study because very few studies have investigated GC metastasis. In the presence of Wnt ligands, stable β-catenin is transported into the nucleus via Rac1 and other factors, where it binds to LEF/TCF transcription factors and recruits additional helper activators for Wnt target genes, such as Cyclin D1, C-Myc, Axin2 and MMP-7, to increase the migration ability and decrease the adhesion ability of cells^33,34^. Studies have shown that miR-18a and miR-19a contribute to tumour metastasis by targeting the negative regulators of Wnt/β-catenin signalling, such as PIAS3 and MXD1^35,36^. Our western blot results confirm that miR-18a and miR-19a increase the expression levels of β-catenin, C-Mycand Axin2. To further enrich the understanding of the mechanism through which miR-18a and miR-19a promote GC metastasis, our data analysis predicted the potential miR-18a and miR-19a targets located in the SMAD2 promoter. SMAD2 is a key negative modulator of Wnt/β-catenin signalling that recruits β-catenin to inhibit its expression and its downstream molecules^22^. The luciferase reporter assays verified the regulation of SMAD2 transcription by miR-18a and miR-19a. Notably, our data suggest that the upregulation of miR-18a and miR-19a significantly promotes C-Myc overexpression, whereas previous studies reported that the transcription of MIR17HG is directly transactivated by C-Myc^37^. This finding indicates a novel finding, namely, the potential existence of a positive feedback loop between MIR17HG and C-Myc.

IRF-1 is a nuclear transcription regulator that has an important role in cancer proliferation, apoptosis and the DNA damage response^26,38^. Mutations and defects of IRF-1 are associated with the development of GC and leukaemia^26,39^. In recent years, the role of IRF-1 in tumour metastasis has gradually attracted attention. For example, various carcinogenic factors, such as miR-383, deltex (DTX)-3-like E3 ubiquitin ligase (DTX3L) and poly (ADP-ribose) polymerase family member 9 (PAPR9), have been shown to promote tumour metastasis and invasion by targeting IRF-1^40,41^. Moreover, IRF-1 is an essential mediator of the interaction between tumour cells and natural killer (NK) cells to regulate immune surveillance in metastatic niches^42^. The IFN-γ/IRF-1/let-7a cluster/EMT pathway has a critical role in regulating the spread of circulating colorectal cancer cells to the liver^43^. However, the role of IRF-1 in GC metastasis has not been reported. Here, our data further support a tumour-suppressive role for IRF-1 in GC metastasis. An analysis of TCGA data revealed that IRF-1 expression was decreased in GC and negatively correlated with MIR17HG expression, which prompted us to further explore the role of IRF-1 in MIR17HG expression. Increased IRF-1 inhibited MIR17HG expression both in vivo and in vitro, and the corresponding inhibited expression of miR-18a and miR-19a reinforced the repression of Wnt/β-catenin signalling. Our exploration of the precise mechanism underlying this phenomenon demonstrated the presence of potential binding sites for IRF-1 on the MIR17HG promoter, and ChIP and luciferase activity assays further clarified the binding site sequence. Thus, IRF-1 has a tumour metastasis-suppressive role in GC by preventing MIR17HG-miR-18a/19a axis mediated Wnt/β-catenin signalling.

This study has important therapeutic implications for GC. Using data sets of GC, we confirmed the promotional effect of MIR17HG on tumour metastasis and its diagnostic significance in GC. More importantly, IRF-1 is increasingly thought to play an important role in the development of malignant tumours^26,41^. Multiple studies have also shown that IRF-1 can be used as a predictive biomarker and therapeutic target for malignant tumours^30,31^. Our in vitro and in vivo studies further support the notion that IRF-1 is likely an important target and biomarker for the inhibition of GC metastasis. Moreover, our results also demonstrate that the type II interferon IFN-γ, which is mediated by IRF-1, can attenuate MIR17HG expression in GC cells and therefore further links the tumour immune response to cancer cell metastasis.

Owing to the large number of identified miRNAs, we could not conduct individual mechanistic studies on these molecules, which is a limitation of our study. Our results showed that miR-19b-1 and miR-92–1 have few correlations with tumour metastasis (Supplementary Table S3). Combined with previous studies of miR-17 and miR-20a in tumour invasion^13–15^, we have not further explored their roles and mechanisms in tumour metastasis, but the potential impact of embedded miRNAs of MIR17HG in the malignant development of GC should be comprehensively investigated. In addition, although the expression of miR-18a and miR-19a affects the growth and proliferation of GC cells, our results indicate that IRF-1 expression does not inhibit GC cell proliferation. Therefore, we did not further investigate the mechanisms of IRF-1 and miR-18a/19a in cell proliferation. There may be other upstream factors that inhibit the activity of miR-18a/19a in promoting cell proliferation, which will be the focus of future investigations.

In summary, our study provides a new understanding of the previously unrecognised mechanisms of MIR17HG in promoting GC metastasis. We first identified that miR-18a and miR-19a coordinately activate GC metastasis by directly targeting SMAD2 and upregulating Wnt/β-catenin signalling. Our study further confirmed that IRF-1 can effectively transcriptionally inhibit the expression of MIR17HG and overcome the promotion of GC metastasis, and these findings underscore the potential clinical relevance of the interactions between IRF-1 and MIR17HG.

## Materials and methods

### Clinical sample collection

A total of 20 pairs of GC tissues and adjacent tissues from patients who underwent surgical treatment at Union Hospital (Wuhan, China) between April and May 2018 were utilised in this study. The diagnosis of GC was confirmed by the original histopathological reports. The tissue samples were individually rapidly frozen at − 80 °C or fixed in formalin for paraffin embedding. All the patients provided written informed consent to participate in this study, which was approved by the Ethics Committee of the Academic Medical Centre of Huazhong University of Science and Technology.

### Cell lines and culture

Human GC cell lines (MKN45, AGS and SGC7901) and a normal gastric epithelial cell line (GES1) were purchased from the BeNa Culture Collection (Beijing, China). All the cells were cultured in Roswell Park Memorial Institute (RPMI) 1640 medium (Gibco, NY, USA) supplemented with 10% foetal bovine serum (FBS) (Sciencell, CA, USA) and 1% penicillin/streptomycin (HyClone, UT, USA) at 5% CO_2_ and 37 °C. The IFN-γ used for the experimental treatment of the cells was purchased from PeproTech (NJ, USA).

### Construction and infection of Lv-IRF-1

The human IRF-1 gene was inserted between the AgeI and EcoRI sites of vector GV308 to obtain Lv-IRF-1. The recombinant lentivirus was propagated in 293 T cells and purified by centrifugation and subsequent dialysis. The viral titres were determined by fluorescence and drug screening, and the recombinant viruses were stored in a virus preservation solution at − 80 °C. The presence of the lentivirus product IRF-1 was confirmed by western blot analysis of infected 293 T cells. A lentivirus comprising the empty vector GV308 (Lv-Null) was constructed as a control. The recombinant lentivirus containing the IRF-1 gene was stably transfected into all GC cell lines according to the manufacturer’s instructions. The screening drug puromycin was added 72 hours after infection, and its concentration was maintained at 5 μg/ml for 24 hours. The expression of IRF-1 was then induced by the addition of Dox (4 µg/mL) to the medium, and the cells were then cultured in this medium for 48 hours and then subjected to additional assays. All the reagents utilised for the experiments described in this section were purchased from GeneChem (Shanghai, China).

### Oligonucleotide construction and transfection

Mimics, inhibitors and NC oligonucleotides for hsa-miR-18a-5p and hsa-miR-19a-3p were purchased from RiboBio (Guangdong, China), and siRNAs targeting IRF-1 were also obtained from RiboBio (Guangdong, China). Once the cells reached 40–50% confluence, they were transfected with the oligonucleotides using Lipofectamine 3000 reagent (Invitrogen, MA, USA) according to the manufacturer’s instructions. The cells were cultured for an additional 48 hours for subsequent assays.

### Cell migration assay

The cell motility was assessed through cell migration assays using transwell chambers (Beaverbio, Jiangsu, China). Suspensions of 8 × 10^4^ cells in 200 μl of serum-free medium (Sciencell, CA, USA) were added to the upper chamber, and medium containing 20% FBS was added to the lower chamber to serve as the chemoattractant. The chambers were incubated at 37 °C in 5% CO_2_ for 24 hours, and the cells in the upper chamber were then removed with cotton swabs. The migrated cells attached to the lower chamber were fixed with 4% paraformaldehyde (Sigma-Aldrich, Darmstadt, Germany), stained with 0.1% crystal violet (Goodbio, Wuhan, China) and quantified by light microscopy. Cells from five random fields were counted.

### Wound-healing assay

The cells were seeded in 12-well plates (Beaverbio, Jiangsu, China) and grown to 90% confluence in complete medium. The artificial wound was prepared by scraping the confluent cell monolayer with a 200-µl pipette tip and then washing with phosphate-buffered saline (PBS, Gibco, NY, USA) to remove the isolated cells. The cells were grown in serum-free medium at 37 °C with 5% CO_2_ for 24 and 48 h. Cell migration was assessed by microscopy and analysed objectively with ImageJ 1.8.0. The wound closure percentages were calculated using the following formula: 1−[24- or 48-hour area/0−hour area]. Three independent assays were photographed and quantified.

### Cell counting kit-8 assay

Cells (1 × 10^3^ per well) were seeded into 96-well plates with 100 μl of RPMI 1640 medium containing 10% FBS and cultured in a humidified incubator at 37 °C for 24, 48 and 72 hours. Then, 10 μl CCK-8 (Meilunbio, Dalian, China) solution was added into each well, and the plate was incubated at 37 °C for 2 hours. A microplate reader was used to detect the absorbance at 450 nm. All experiments were performed at least three times independently.

### Colony formation assay

Cells (1 × 10^3^ per well) were seeded into six-well plates and cultured in a humidified incubator at 37 °C for 15 days, with medium changes every 3 days. Then, the colonies were stained with 0.1% crystal violet for 30 min after fixation with methanol for 10 min, and the colonies were counted. All experiments were performed at least three times independently.

### Cell cycle analysis

After transfection, cells (1 × 10^6^ per well) were collected and washed twice with PBS and then fixed with 70% ethanol for 4 hours on ice. Then, the cell cycle was analysed using a cell cycle detection kit (KGA512, KeyGEN, Nanjing, China) following the manufacturer’s instructions and was detected using flow cytometry (Beckman Coulter, CA, USA). All experiments were performed at least three times independently.

### Western blot analysis

Protein lysates from the cultured cells were prepared with radioimmunoprecipitation buffer (Sigma-Aldrich, Darmstadt, Germany) containing proteinase and phosphatase inhibitors, and western blotting was then performed as previously described^44,45^. Primary antibodies against IRF-1 (Abcam, ab186384, Cambridge, UK, 1:1000 dilution), β-catenin (Proteintech, 51067–2-AP, Wuhan, China, 1:750 dilution), C-Myc (Proteintech, 10828–1-AP, Wuhan, China, 1:750 dilution), SMAD2 (Proteintech, 12570–1-AP, Wuhan, China, 1:750 dilution), Axin2 (Proteintech, 20540–1-AP, Wuhan, China, 1:750 dilution), Stat1 (Proteintech, 10144–2-AP, Wuhan, China, 1:750 dilution) and glyceraldehyde 3-phosphate dehydrogenase (GAPDH; Proteintech, 10494–1-AP, Wuhan, China, 1:5000 dilution) were used, and GAPDH was used for normalisation of the protein levels. Signal detection was performed using an enhanced chemiluminescence detection kit (Meilunbio, Dalian, China).

### Quantitative real-time PCR

The total RNA from cultured cells or tumour tissues was extracted using the TRIzol reagent kit (Takara, Dalian, China). A quantitative analysis of miRNA and mRNA expression was performed using the StepOne Plus Real-Time PCR System (Applied Biosystems). For miRNA detection, TaqMan miRNA assays (Life Technologies) and reverse transcription real-time PCR (Takara, Dalian, China) were performed according to the manufacturers’ instructions; U6 small nuclear RNA was used as the internal control. For mRNA detection, complementary DNA was generated with the PrimeScript RT Reagent kit (Takara, Dalian, China), and qRT-PCRs were performed using SYBR Premix Ex Taq (Takara, Dalian, China) according to the manufacturer’s recommended protocol. The GAPDH gene was used as an internal control. The primers for all the genes were synthesised by RiboBio (Guangdong, China) (Supplementary Tables S7 and S8). The relative expression levels of the genes were calculated using the 2^−ΔΔCq^ method.

### ChIP

The ChIP assay was performed using the SimpleChIP Enzymatic Chromatin IP Kit (CST, #9003, MA, USA) according to the manufacturer’s recommended protocol. Chromatin extracts containing DNA fragments (~ 150~900 bp) were immunoprecipitated using 4 μg of a monoclonal anti-IRF-1 antibody (Santa, sc-74530, CA, USA), 10 μg of a rabbit monoclonal histone H3 antibody (positive control, CST, MA, USA) or 2 μg of a rabbit IgG (whole molecule, NC, CST, MA, USA). The purified DNA was subjected to qPCR (the primers are shown in Supplementary Table S9) to amplify the binding sites of the MIR17HG promoter region. The data are expressed as relative enrichments normalised to the negative control IgG.

### Luciferase reporter assay

For luciferase reporter assays of SMAD2 3′-UTR, the indicated GC cells were co-transfected with miR-18a or miR-19a mimic or negative control mimic and with the indicated dual luciferase reporter constructs containing the 3′-UTR wild-type or mutant using Lipofectamine 3000 (Thermo Fisher Scientific). For the luciferase reporter assay for measuring promoter activities, the indicated cells plated in 24-well plates were co-transfected with MIR17HG luciferase reporter constructs and IRF-1 plasmids using the Lipofectamine 3000 reagent. The firefly and Renilla luciferase activities after 48 hours were measured using a dual luciferase reporter assay (Promega, Madison, WI, USA) according to the manufacturer’s recommended protocol. The luciferase readings were normalised to the Renilla luciferase activity and are presented as relative luciferase activities. All the assays were performed three times in triplicate.

### In vivo metastasis model

Six-week-old female BALB/c nude mice were purchased from HFK Bio-Technology (Beijing, China) and maintained under specific pathogen-free conditions at the Experiment Animal Center of Huazhong University of Science and Technology. Suspensions of the corresponding cells were injected into the mouse tail veins (5 × 10^6^ tumour cells/150 μl of PBS per mouse; four mice per group). After 10 days, two groups (SGC7901/Lv-Null and SGC7901/Lv-IRF-1) of mice were treated with 4 mg/mL Dox via their drinking water, and the water was changed every 3 days. After 20 days of feeding, the mice were sacrificed according to institutional ethics guidelines. The post-mortem examinations and recordings included the mouse body weight, metastasis locations and number of nodules. The tumour tissues were then individually frozen in optimal cutting temperature media or fixed in formalin for paraffin embedding. All the experiments were conducted using protocols approved by the Animal Research Committee of the Academic Medical Centre at Huazhong University of Science and Technology. All the experimental procedures were performed in accordance with the guidelines of the Institutional and Animal Care and Use Committees.

### IHC

Paraffin-embedded mouse tumour metastatic tissues and clinical GC tissues were cut into 5-μm-thick sections, placed on glass slides and stained with H&E or subjected to IHC analysis. The tissue sections were deparaffinized and subjected to antigen retrieval using 0.01 m citric acid buffer (pH 6.0) at 95m °C for 15 min and incubated overnight at 4 °C with primary antibodies against IRF-1 (Abcam, ab186384, Cambridge, UK, 1:50 dilution), β-catenin (Proteintech, 51067–2-AP, Wuhan, China, 1:50 dilution) and C-Myc (Proteintech, 10828–1-AP, Wuhan, China, 1:50 dilution). After three washes with Tris-buffered saline, the sections were incubated with horseradish peroxidase-conjugated Affinipure goat anti-rabbit IgG (Abcam, ab6795, Cambridge, UK, 1:100 dilution) for 1 hour at room temperature. The IHC results were scored by two independent observers.

### Data set acquisition and statistical analysis

Raw GC patient data containing RNA sequencing (RNA-Seq), miRNA sequencing (miRNA-Seq) and clinical information were obtained from the Broad Institute TCGA Genome Data Analysis Center (http://gdac.broadinstitute.org/runs/analyses__latest/reports/cancer/STAD/). The GC miRNA expression array data used as a validation data set were acquired from the NCBI Gene Expression Omnibus (GEO) (GEO accession: GSE93415 and GSE23739)^46^.

The statistical analyses were performed using SPSS 24.0 and R (version 3.3.1). The statistical significances between the groups are expressed as *P* values, and *P* values < 0.05 are considered to indicate statistical significance. The gene expression abundances of the data sets were log_2_-transformed. The genes that met the following criteria were treated as differentially expressed genes: |log_2_(fold change) | > 1 and a false discovery rate <0.05. Pearson correlation coefficients were used to measure the correlation between mRNA and miRNA expression. Clinical correlation analyses of the TCGA data were performed by chi-square test (excluding T1 patients), and the median expression values of the genes were set as the cutoff values between high and low expression. A ROC curve analysis was performed to determine the predictive value of the parameters, and the AUC was detected. The experimental results were analysed through two-tailed unpaired or paired Student’s *t* test or Pearson correlation coefficients according to the type of experiment performed.

## Supplementary information

Supplementary Information

Supplementary Figure S1

Supplementary Figure S2

Supplementary Figure S3

Supplementary Figure S4

Supplementary Figure S5

Supplementary Figure S6

Supplementary Table S4
