# Supplementary material for: MIR17HG-miR-18a/19a axis, regulated by interferon regulatory factor-1, promotes gastric cancer metastasis via Wnt/β-catenin signalling
Source: Cell Death Dis. 2019 Jun 11;10(6):454. doi: 10.1038/s41419-019-1685-z (PMC6560107; doi:10.1038/s41419-019-1685-z)
Supplement: Supplementary file 1 — Supplementary Information [file 41419_2019_1685_MOESM1_ESM.docx]

**Supplementary Information**

**MIR17HG-miR18a/19a axis, regulated by interferon regulatory factor-1, promotes gastric cancer metastasis via Wnt/β-catenin signalling**

Jingsheng Yuan^1, *^, Lulu Tan^1, *^, Zhijie Yin^1^, Wenzhong Zhu^1^, Kaixiong Tao^1^, Guobing Wang^1^, Wenjia Shi^2^ and Jinbo Gao^1^

**Table of contents**

**Supplementary Figures S1-6**

**Supplementary Tables S1-9**

**Supplementary Figure S1: Expression profile of MIR17HG in the TCGA dataset.** (A) Genomic organization and primary transcript structures of human MIR17HG. (B) The differentially expressed mRNAs in 238 GC samples and 33 adjacent gastric mucosal tissues are visualized in a volcano plot. The red and green colours indicate high and low expression (|log2(fold change) | >1, FDR <0.05), respectively. (C) Scatter plots of MIR17HG versus its derived miRNA expression in the TCGA GC dataset. P values and Pearson correlation coefficients (R) are shown.

**Supplementary Figure S2: Knockdown of miR-18a and miR-19a inhibits GC cell metastasis.**

(A) At 48 hours after transfection, the expression of miR-18a and miR-19a in the MKN45 and AGS cell lines was examined by qRT-PCR, with U6 snRNA serving as the internal control. (B) Wound-healing assay and (C) migration assay of MKN45 and AGS cells treated with an NC inhibitor, a miR-18a inhibitor, a miR-19a inhibitor and an inhibitor mixture. All of the above experiments were independently performed in triplicate (N=3). The data are presented as the means ± SDs. *P<0.05, as demonstrated by paired Student’s t-test.

**Supplementary Figure S3: Effect of miR-18a and miR-19a on the proliferation and cell cycle of GC cells.**

(A) The CCK-8 assay and (B) colony assay results revealed that transfection of the miR-18a mimic, miR-19a mimic and mimic mixture in GC cells increased cellular progression. (C) The CCK-8 assay and (D) colony assay results revealed that transfection of the miR-18a inhibitor, miR-19a inhibitor and inhibitor mixture in GC cells inhibited cellular progression. (E) GC cells treated with mimics or inhibitors were analysed for the cell cycle distribution using flow cytometry. All of the above experiments were independently performed in triplicate (N=3). *P<0.05, as determined by paired Student’s t-test. The data are presented as the means ± SDs.

**Supplementary Figure S4: miR-18a/19a drive GC cell metastasis by regulating the Wnt/β-catenin signalling pathways.**

(A) Western blot analysis of β-catenin, C-Myc and Axin2 in MKN45 and AGS cells treated with an NC inhibitor and a miR-18a/19a inhibitor mixture. (B) Western blot analysis of E-cadherin, N-cadherin and vimentin in MKN45 and AGS cell lines treated with NC mimic and miR-18a/19a mimic mixture. All of the above experiments were independently performed in triplicate (N=3). (C) Venn diagram of the potential targets of miR-18a/19a and the putative gene targets of the six miRNAs enriched in the Wnt/β-Catenin signalling pathway. (D) After treatment of MKN45 and AGS cells with NC mimic and miR-18a/19a mimic mixture, the expression of seven putative targets of miR-18a/19a was measured by qRT-PCR analysis. Three independent experiments were performed in triplicate (N=3). *P<0.05, as determined by paired Student’s t-test. The data are presented as the means ± SDs.

**Supplementary Figure S5: IRF-1 is negatively correlated with MIR17HG expression.**

(A) IRF-1 expression in different tissues. The data are presented as the means ± standard deviations (SDs). STAD indicates gastric adenocarcinoma. The data were obtained from the Broad Institute TCGA Genome Data Analysis Center (2016) (http://gdac.broadinstitute.org/runs/analyses__latest/reports/cancer/STAD/). (B) Scatter plots of IRF-1 versus MIR17HG expression in the TCGA GC data set. P values and R values are shown. (C) Scatter plots of IRF-1 versus miR-18a and miR-19a expression in the TCGA GC data set. P values and R values are shown. (D) The correlation between IRF-1 versus miR-18a and miR-19a was analysed among 20 GC sample tissues. P values and R values are shown. Three independent experiments were performed in triplicate (N=3). (E) CCK-8 assays and (F) colony assays of Dox-treated and Dox-untreated Lv-IRF-1 MKN45 cells were performed. Three independent experiments were performed in triplicate (N=3). *P<0.05, as determined by paired Student’s t-test. The data are presented as the means ± SDs.

**Supplementary Figure S6: IRF-1 regulates MIR17HG expression in vitro and in vivo.**

(A) After 48 hours of induction with or without Dox, SMAD2 expression in MKN45, AGS and SGC7901 cells transfected with Lv-IRF-1 was analysed by western blotting. (B) Wound-healing assay and (C) migration assay of MKN45 and AGS after IRF-1 knockdown. (D) Immunohistochemistry images of IRF-1, β-catenin and C-Myc staining in four representative human GC specimens with high and low miR-18a expression. Samples 1 to 4 are from Supplementary Figure S2G. Scale bars, 200 μm. (E) The expression level of Stat1 in AGS, MKN45 and SGC7901 cells was assessed by western blotting. (F) AGS cells were treated with the indicated amount of IFN-γ for 48 hours, and the expression of IRF-1 was then determined by western blotting. All the above experiments were independently performed in triplicate (N=3). *P<0.05, as determined by paired Student’s t-test. The data in A, B, E and F are presented as the means ± SDs.

**Supplementary Table S1**

| **Clinical features** | **n** | **MIR17HG** |  | **P-value** |
| --- | --- | --- | --- | --- |
|  |  | **Low expression** | **High expression** |  |
| **Age (years)** |  | | | |
| **<=65** | 103 | 51 | 52 | 0.929 |
| **>65** | 88 | 43 | 45 |  |
| **gender** |  | | | |
| **female** | 74 | 40 | 34 | 0.29 |
| **male** | 119 | 55 | 64 |  |
| **histological type** |  | | | |
| **stomach adenocarcinoma** | 128 | 66 | 62 | 0.414 |
| **intestinal adenocarcinoma** | 64 | 29 | 35 |  |
| **histologic grade** |  | | | |
| **G1+G2** | 52 | 22 | 30 | 0.322 |
| **G3** | 137 | 69 | 68 |  |
| **Metastasis** |  | | | |
| **M0** | 176 | 101 | 75 | 0.084 |
| **M1** | 9 | 2 | 7 |  |
| **Lymph node status** |  | | | |
| **N0** | 52 | 33 | 19 | *0.007 |
| **N1+2+3** | 140 | 58 | 82 |  |
| **Microsatellite instability** |  | | | |
| **MSS** | 98 | 41 | 57 | 0.881 |
| **MSI** | 42 | 17 | 25 |  |

**Supplementary Table S1: Correlation of MIR17HG expression with clinicopathological parameters. *P<0.05 by chi-square test. MSS, microsatellite stability; MSI, microsatellite instability.**

**Supplementary Table S2**

| **Clinical features** | **n** | **mir-18a** |  | **P-value** | **mir-19a** |  | **P-value** |
| --- | --- | --- | --- | --- | --- | --- | --- |
|  |  | Low expression | High expression |  | Low expression | High expression |  |
| **Age (years)** |  | | | | | | |
| **<=65** | 126 | 64 | 62 | 0.332 | 69 | 57 | 0.116 |
| **>65** | 108 | 48 | 60 |  | 48 | 60 |  |
| **gender** |  | | | | | | |
| **female** | 87 | 46 | 41 | 0.238 | 41 | 46 | 0.499 |
| **male** | 147 | 66 | 81 |  | 76 | 71 |  |
| **histological type** |  | | | | | | |
| **stomach adenocarcinoma** | 130 | 72 | 58 | *0.010 | 72 | 58 | 0.066 |
| **intestinal adenocarcinoma** | 104 | 40 | 64 |  | 45 | 59 |  |
| **histologic grade** |  | | | | | | |
| **G1** | 72 | 25 | 47 | *0.011 | 33 | 39 | 0.472 |
| **G2+G3** | 157 | 83 | 74 |  | 80 | 77 |  |
| **Metastasis** | | | | | | | |
| **M0** | 221 | 114 | 107 | *0.018 | 111 | 110 | 0.564 |
| **M1** | 12 | 2 | 10 |  | 5 | 7 |  |
| **Lymph node status** |  | | | | | | |
| **N0** | 56 | 32 | 24 | *0.040 | 37 | 19 | *0.007 |
| **N1+2+3** | 176 | 73 | 103 |  | 80 | 96 |  |
| **Microsatellite instability** |  | | | | | | |
| **MSS** | 163 | 83 | 80 | 0.156 | 91 | 72 | *0.007 |
| **MSI** | 71 | 29 | 42 |  | 26 | 45 |  |

**Supplementary Table S2: Correlation of miR-18a and miR-19a expression with clinicopathological parameters. *P<0.05 by chi-square test. MSS, microsatellite stability; MSI, microsatellite instability.**

**Supplementary Table S3**

| **Clinical features** | | **Metastasis** | | **P-value** | **Lymph node status** | | **P-value** |
| --- | --- | --- | --- | --- | --- | --- | --- |
|  |  | **M0** | **M1** |  | **N0** | **N1+2+3** |  |
|  |  | 221 | 12 |  | 56 | 176 |  |
| **miR-17** | **Low** | 122 | 2 | *0.009 | 34 | 69 | *0.047 |
|  | **High** | 99 | 10 |  | 22 | 107 |  |
| **miR-20a** | **Low** | 107 | 4 | 0.304 | 39 | 80 | *0.026 |
|  | **High** | 114 | 8 |  | 17 | 96 |  |
| **miR-19b-1** | **Low** | 132 | 4 | 0.068 | 32 | 89 | 0.563 |
|  | **High** | 89 | 8 |  | 24 | 87 |  |
| **miR-92-1** | **Low** | 119 | 5 | 0.406 | 35 | 79 | 0.105 |
|  | **High** | 102 | 7 |  | 21 | 97 |  |

**Supplementary Table S3: Correlation of miR-17, miR-20a, miR-19b-1 and miR-92-1 expression with tumor metastasis and lymph node status. *P<0.05 by chi-square test.**

**Supplementary Table S4**

**The potential targets of six miRNAs derived from MIR17HG. Data came from DIANA(http://diana.imis.athena-innovation.gr/DianaTools/index.php?r=microT _CDS/index). ^16, 17^**

**Supplementary Table S5**

| **Term** | **FDR** | **Fold Enrichment** | **Bonferroni** | **Benjamini** |
| --- | --- | --- | --- | --- |
| hsa05200:Pathways in cancer | 4.96E-46 | 8.501671912 | 4.58E-47 | 4.58E-47 |
| hsa04510:Focal adhesion | 1.69E-14 | 6.528647087 | 1.56E-15 | 3.89E-16 |
| hsa04012:ErbB signaling pathway | 9.43E-11 | 9.427141268 | 8.70E-12 | 1.24E-12 |
| **hsa04310:Wnt signaling pathway** | 4.42E-08 | 5.974684896 | 4.08E-09 | 3.40E-10 |
| hsa04722:Neurotrophin signaling pathway | 6.18E-07 | 6.283493757 | 5.71E-08 | 3.57E-09 |
| hsa04912:GnRH signaling pathway | 7.31E-05 | 6.276744569 | 6.75E-06 | 3.75E-07 |
| hsa04062:Chemokine signaling pathway | 8.52E-05 | 4.385889253 | 7.86E-06 | 4.14E-07 |
| hsa04540:Gap junction | 1.70E-04 | 6.450706778 | 1.57E-05 | 7.85E-07 |
| hsa04810:Regulation of actin cytoskeleton | 7.79E-04 | 3.814703676 | 7.19E-05 | 3.42E-06 |
| hsa04115:p53 signaling pathway | 0.004709955 | 6.633657495 | 4.35E-04 | 1.98E-05 |
| hsa04010:MAPK signaling pathway | 0.005252379 | 3.225353389 | 4.85E-04 | 2.11E-05 |
| hsa04070:Phosphatidylinositol signaling system | 0.010294461 | 6.095793374 | 9.50E-04 | 3.80E-05 |
| hsa04910:Insulin signaling pathway | 0.020648912 | 4.252688172 | 0.00190396 | 7.33E-05 |

**Supplementary Table S5: Pathway enrichment analyses in the putative targets of six miRNAs derived from MIR17HG. Data came from DAVID 6.7 (https://david.ncifcrf.gov/summary.jsp). ^18, 19^**

**Supplementary Table S6**

| **Ensembl** | **Factor name** |
| --- | --- |
| ENSG00000138378 | STAT4 |
| ENSG00000172216 | C/EBPbeta |
| ENSG00000100811 | YY1 |
| ENSG00000112592 | TFIID |
| ENSG00000049768 | FOXP3 |
| ENSG00000128709 | HOXD9 |
| ENSG00000128710 | HOXD10 |
| ENSG00000100219 | XBP-1 |
| ENSG00000148737 | TCF-4 |
| ENSG00000135100 | HNF-1A |
| ENSG00000263001 | TFII-I |
| ENSG00000137203 | AP-2alpha |
| ENSG00000196092 | PAX-5 |
| ENSG00000184895 | SRY |
| ENSG00000091831 | ESR1 |
| ENSG00000141510 | TP53 |
| ENSG00000125347 | IRF-1 |
| ENSG00000102145 | GATA-1 |
| ENSG00000136997 | c-Myc |
| ENSG00000185591 | Sp1 |
| ENSG00000134954 | c-Ets-1 |
| ENSG00000168310 | IRF-2 |
| ENSG00000129514 | HNF-3alpha |

**Supplementary Table S6: Possible transcription factor binding sites within the 2.0 kb promoter region of MIR17HG were predicted using the PROMO^21, 22^ and cross-aligned with the JASPAR database.^23^**

**Supplementary Table S7**

| **Primer name** | **Primer sequence** |
| --- | --- |
| RT-hsa-miR-17-5p | GTCGTATCCAGTGCAGGGTCCGAGGTATTCGCACTGGATACGACCTACCT |
| hsa-miR-17-5p-PF | CGCCCAAAGTGCTTACAGT |
| RP2 | CAGTGCAGGGTCCGAGGTAT |
| RT-hsa-miR-18a-5p | GTCGTATCCAGTGCAGGGTCCGAGGTATTCGCACTGGATACGACctatct |
| hsa-miR-18a-5p-F | CCAAGGTAAGGTGCATCTAGTG |
| RP2 | CAGTGCAGGGTCCGAGGTAT |
| RT-hsa-miR-19a-3p | GTCGTATCCAGTGCAGGGTCCGAGGTATTCGCACTGGATACGACtcagtt |
| hsa-miR-19a-3p-F | CCGAGTGTGCAAATCTATGCAA |
| RP2 | CAGTGCAGGGTCCGAGGTAT |
| RT-hsa-miR-20a-5p | GTCGTATCCAGTGCAGGGTCCGAGGTATTCGCACTGGATACGACctacct |
| hsa-miR-20a-5p-F | GCCGGTAAAGTGCTTATAGTGC |
| RP2 | CAGTGCAGGGTCCGAGGTAT |
| RT-hsa-miR-19b-1-5p | GTCGTATCCAGTGCAGGGTCCGAGGTATTCGCACTGGATACGACgctgga |
| hsa-miR-19b-1-5p-F | GCGAGAGTTTTGCAGGTTTG |
| RP2 | CAGTGCAGGGTCCGAGGTAT |
| RT-hsa-miR-92a-1-5p | GTCGTATCCAGTGCAGGGTCCGAGGTATTCGCACTGGATACGACagcatt |
| hsa-miR-92a-1-5p-F | GCCTGAAGGTTGGGATCGGTTGC |
| RP2 | CAGTGCAGGGTCCGAGGTAT |
| RT-U6-2 | CGAATTTGCGTGTCATCCT |
| U6-2-F | CTCGCTTCGGCAGCACATA |
| U6-2-R | CGAATTTGCGTGTCATCCT |

**Supplementary Table S7: List of the sequences of the primers used for miRNAs.**

**Supplementary Table S8**

| **Primer name** | **Primer sequence** |
| --- | --- |
| H-IRF1-F | GAAAAGCATAACACCAATCCCA |
| H-IRF1-R | AAGCCCCTCAGCCAAAGC |
| GAPDH-F | AATCCCATCACCATCTTCCAG |
| GAPDH-R | GAGCCCCAGCCTTCTCCAT |

**Supplementary Table S8: List of the sequences of the primers used for IRF-1.**

**Supplementary Table S9**

| **Primer name** | **Primer sequence** |
| --- | --- |
| Site-1-F | GCTTCAAATTCATTTGGGATT |
| Site-1-R | AGGATTTTGGAAGACGCAAA |
| Site-2-F | AGCTTCAAATTCATTTGGGATT |
| Site-2-F | AGGATTTTGGAAGACGCAAA |

**Supplementary Table S9: List of the sequences of the primers surrounding the putative binding sites for IRF-1 from MIR17HG gene promoter region.**
