# Supplementary figures and images for: MIR17HG-miR-18a/19a axis, regulated by interferon regulatory factor-1, promotes gastric cancer metastasis via Wnt/β-catenin signalling
Source: Cell Death Dis. 2019 Jun 11;10(6):454. doi: 10.1038/s41419-019-1685-z (PMC6560107; doi:10.1038/s41419-019-1685-z)

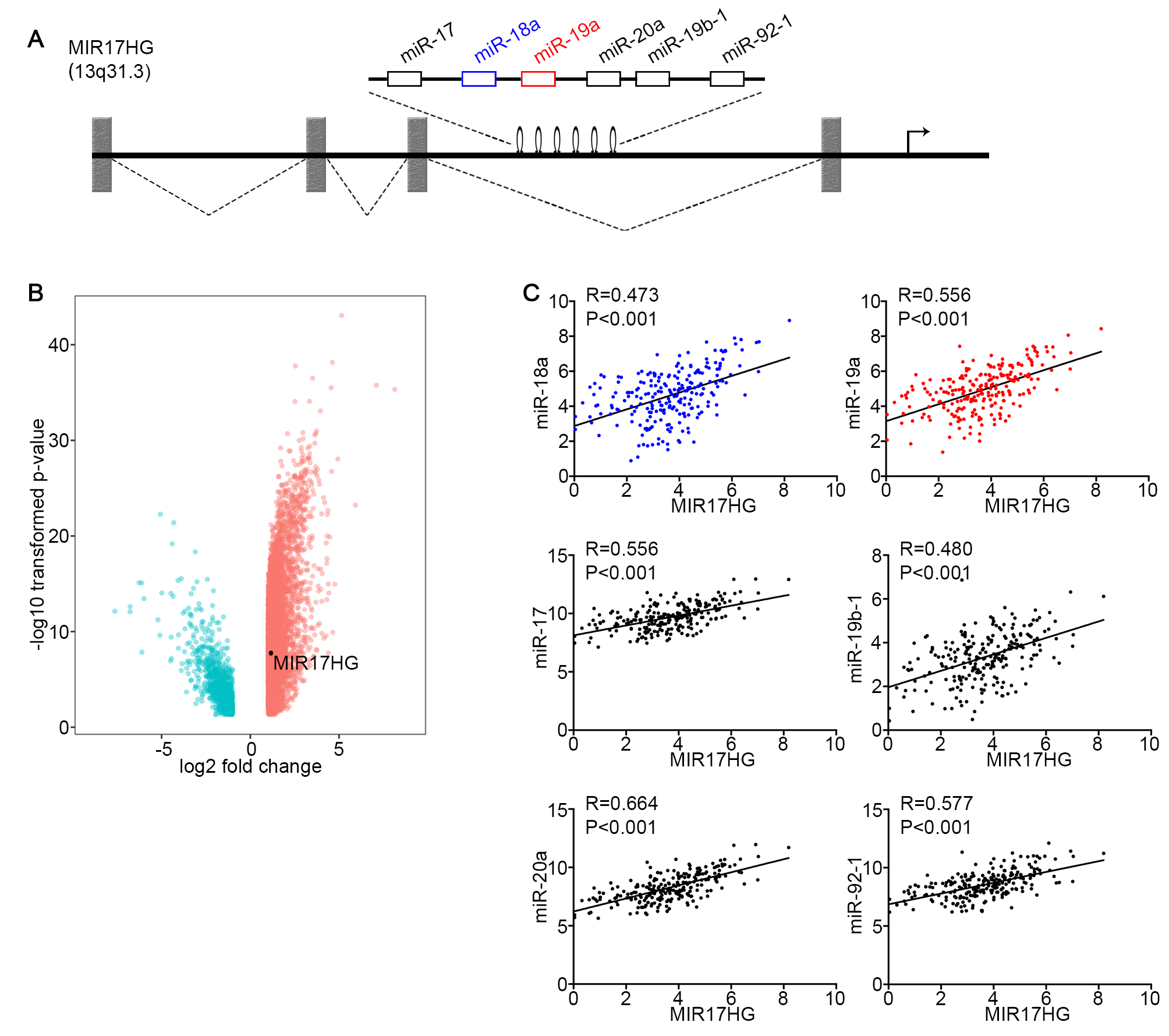

Supplement: Supplementary file 2 — Supplementary Figure S1 [file 41419_2019_1685_MOESM2_ESM.tif]

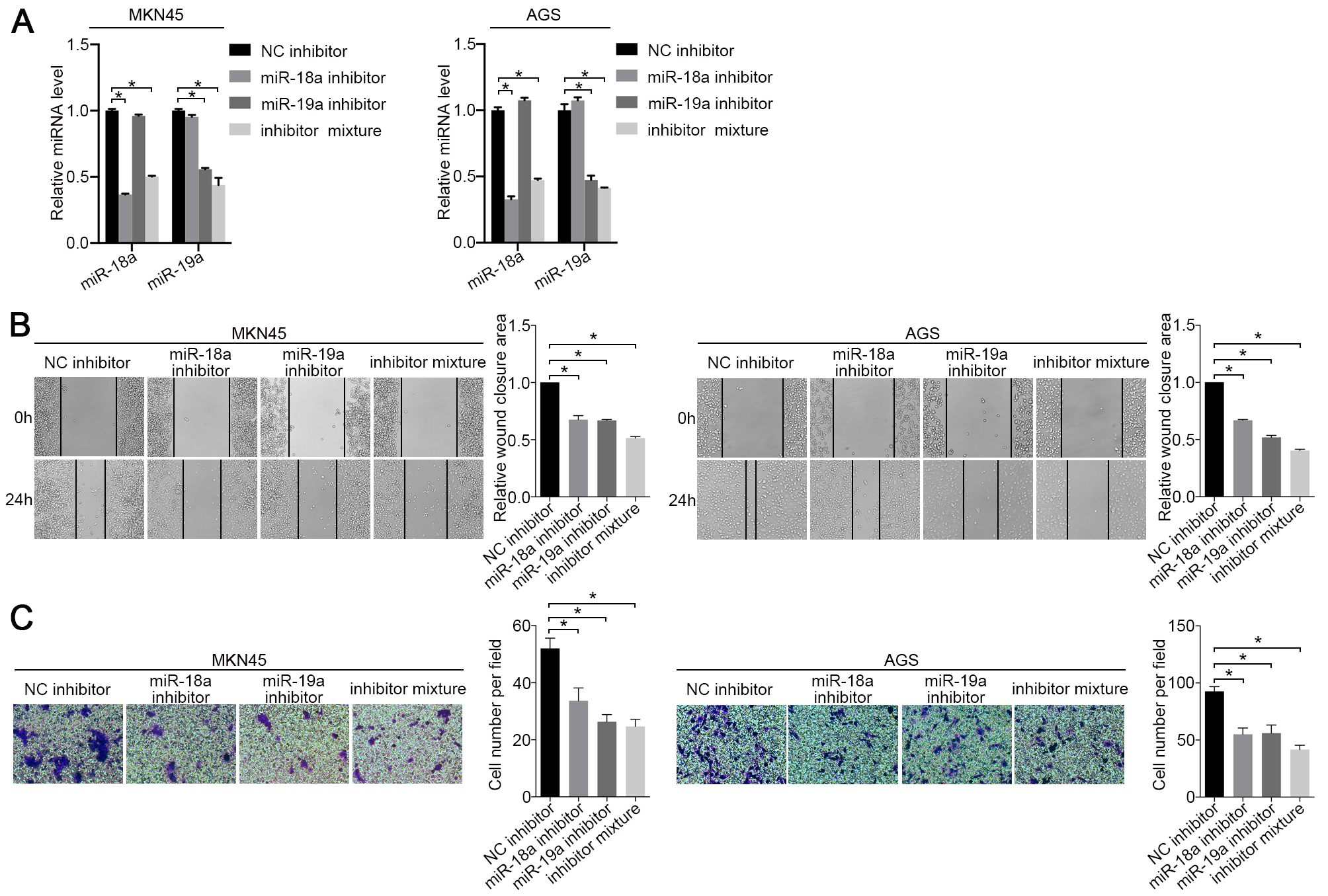

Supplement: Supplementary file 3 — Supplementary Figure S2 [file 41419_2019_1685_MOESM3_ESM.tif]

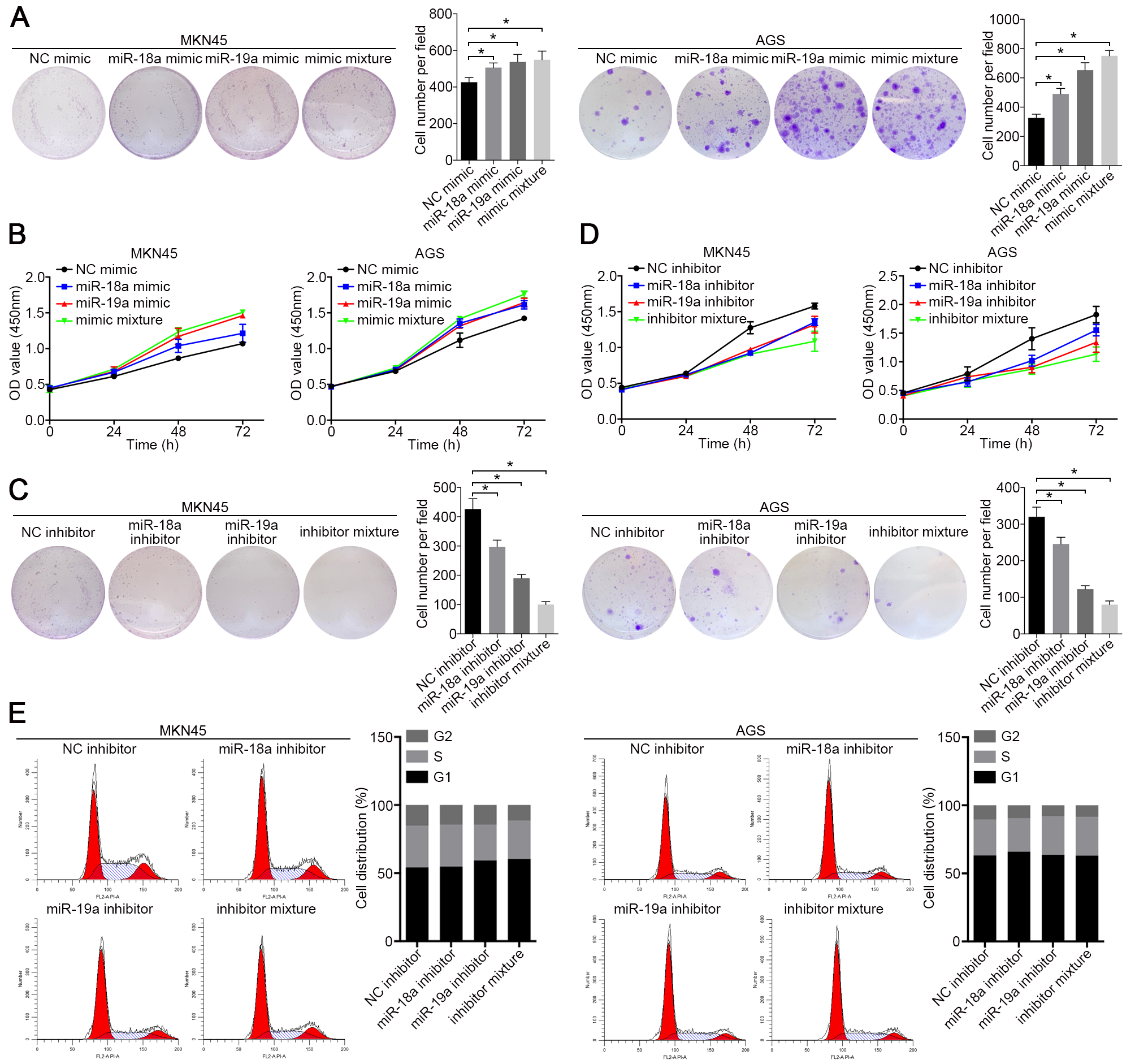

Supplement: Supplementary file 4 — Supplementary Figure S3 [file 41419_2019_1685_MOESM4_ESM.tif]

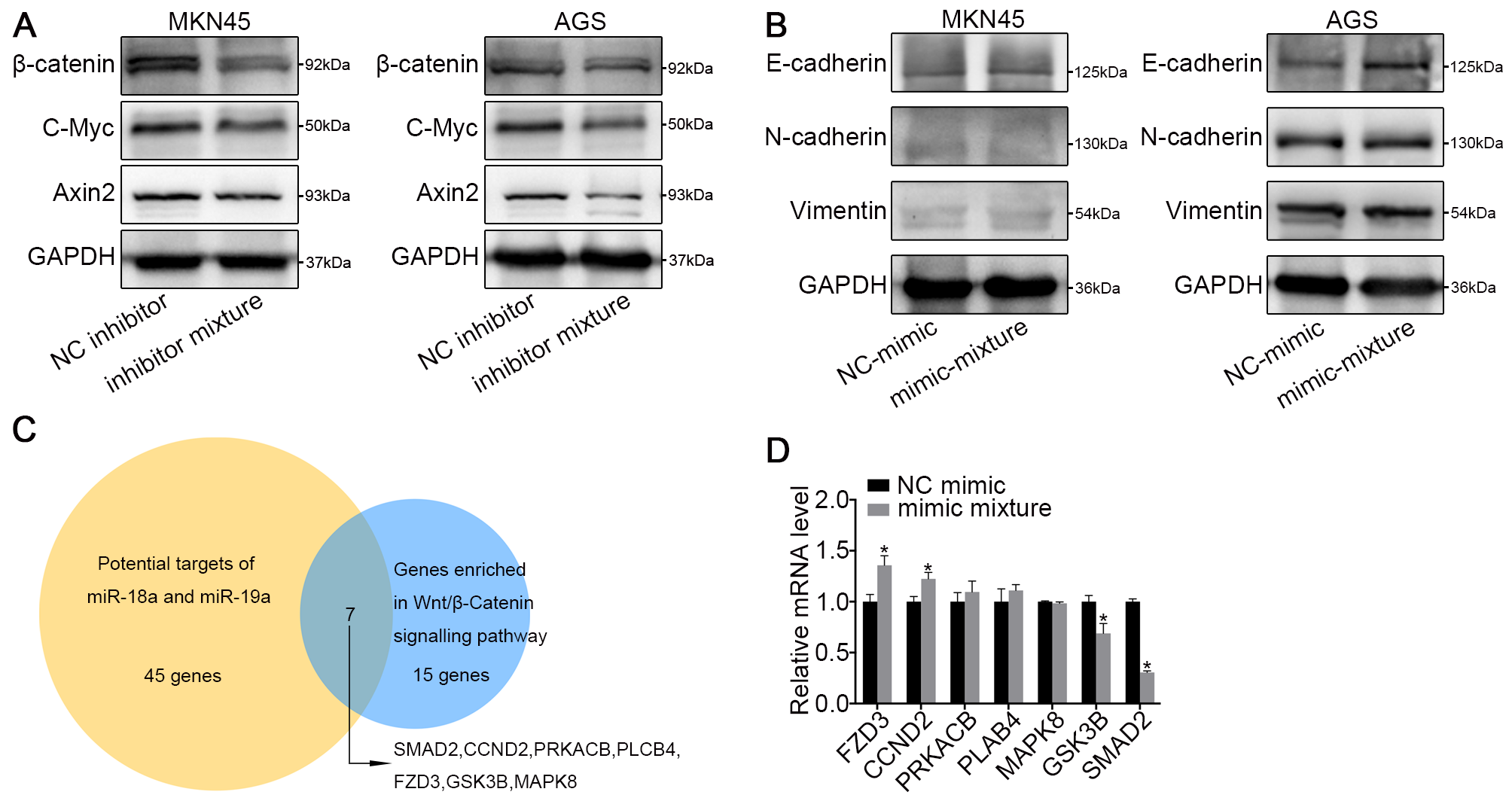

Supplement: Supplementary file 5 — Supplementary Figure S4 [file 41419_2019_1685_MOESM5_ESM.tif]

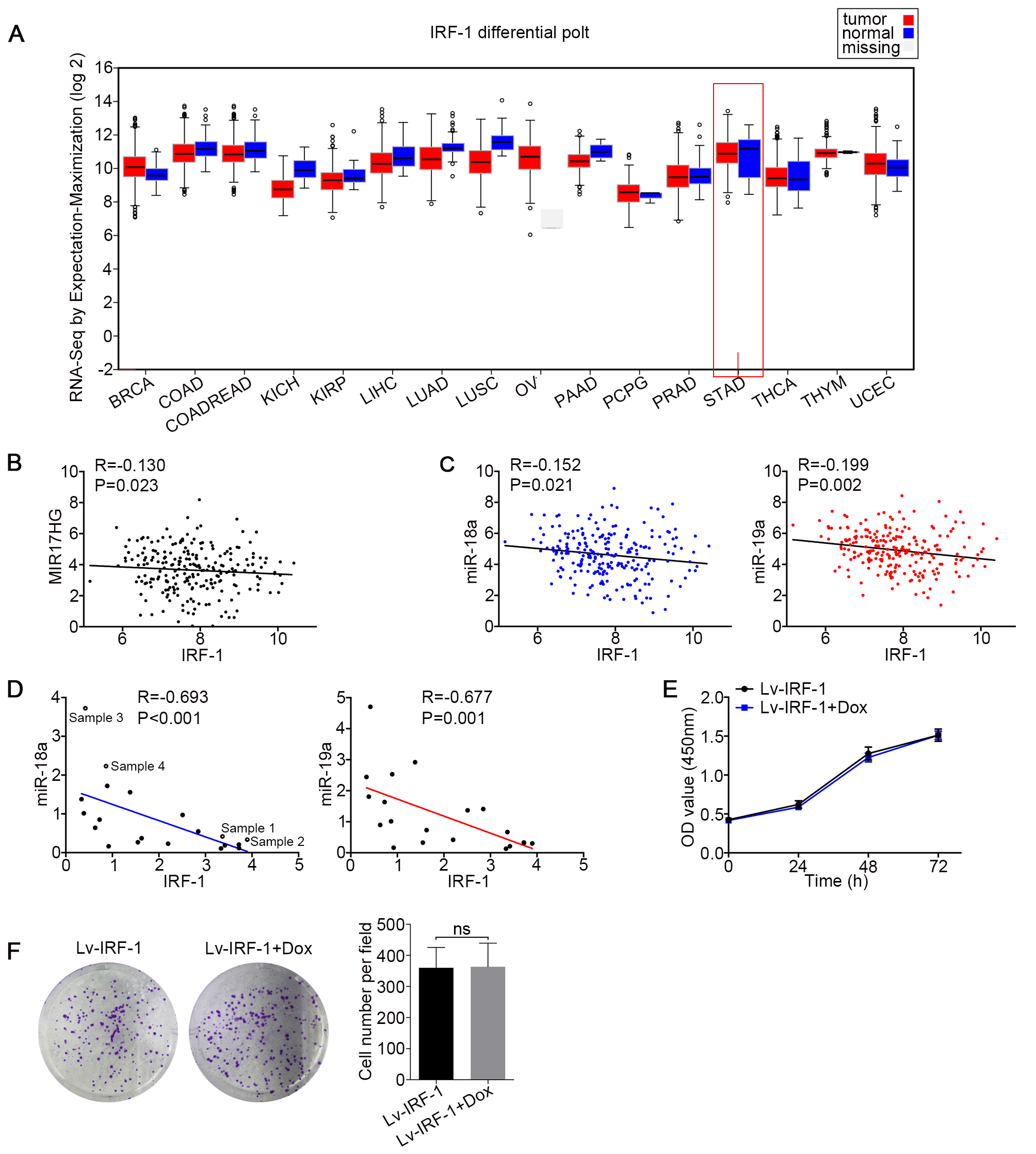

Supplement: Supplementary file 6 — Supplementary Figure S5 [file 41419_2019_1685_MOESM6_ESM.tif]

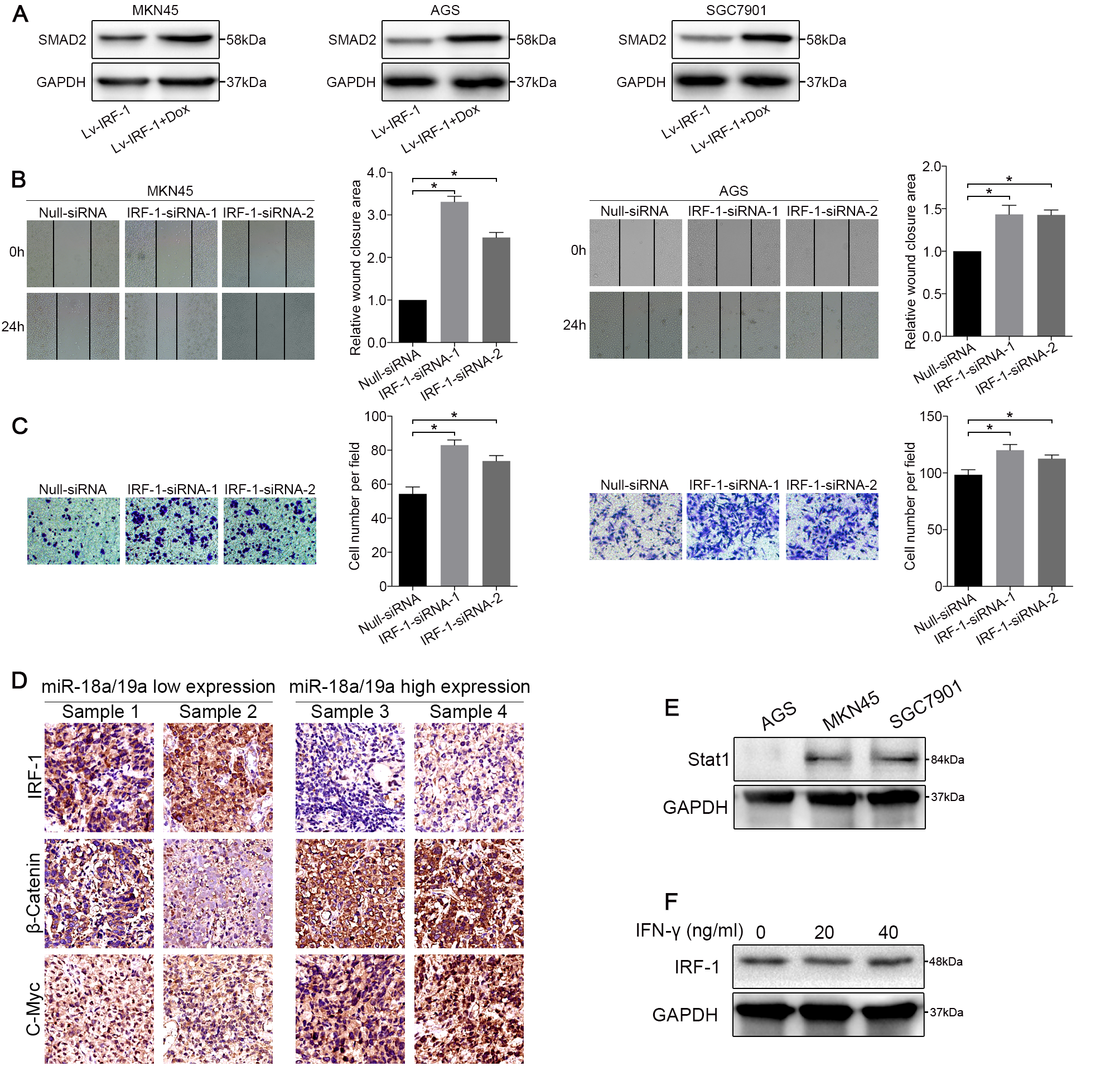

Supplement: Supplementary file 7 — Supplementary Figure S6 [file 41419_2019_1685_MOESM7_ESM.tif]
